# Global, regional, and national burden of ischemic stroke attributable to active smoking, 1990–2021

**DOI:** 10.18332/tid/194697

**Published:** 2024-11-08

**Authors:** Meng Pang, Shuai Hou, Xiaoshuang Xia, Gang Wang, Yanqiang Wang, Lin Wang, Xin Li

**Affiliations:** 1 Department of Neurology, The Second Hospital of Tianjin Medical University, Tianjin, China; 2 Department II of Neurology, The Affiliated Hospital of Shandong Second Medical University, Weifang, Shandong, China; 3 Emergency Department, The Affiliated Hospital of Shandong Second Medical University, Weifang, Shandong, China; 4 Department of Geriatrics, The Second Hospital of Tianjin Medical University, Tianjin, China

**Keywords:** ischemic stroke, active smoking, disease burden, SDI, epidemiology

## Abstract

**INTRODUCTION:**

Ischemic stroke is a major global health issue, with active smoking identified as a key modifiable risk factor. This study examines the burden of ischemic stroke due to active smoking from 1990 to 2021, across different sociodemographic contexts.

**METHODS:**

Data from the Global Burden of Disease (GBD) 2021 database were used to extract information on mortality and disability-adjusted life years (DALYs) attributable to active smoking-related ischemic stroke. Countries and regions were categorized by the sociodemographic index (SDI) into five levels. Statistical analyses were conducted using R Studio, including the calculation of estimated annual percentage change (EAPC) and joinpoint regression models.

**RESULTS:**

In 2021, there were 342674 deaths globally due to ischemic stroke caused by active smoking, with an age-standardized mortality rate (ASMR) of 4.06 and a population-attributable fraction (PAF) of 9.54%. The number of deaths increased by 35.59% from 1990 to 2021, with males aged ≥70 years experiencing the largest increase. The global age-standardized DALY rate in 2021 was 98.29, with an overall increase in DALYs by 33.55% from 1990. Regional analysis revealed significant disparities, with the middle SDI region reporting the highest number of deaths and DALYs, while the high SDI region reported the lowest. Geographically, East Asia had the highest burden in 2021. Nationally, China had the highest number of deaths and DALYs due to smoking-related ischemic stroke.

**CONCLUSIONS:**

This study highlights the significant global burden of ischemic stroke attributable to active smoking and the critical need for targeted smoking cessation programs and stroke prevention strategies. Despite overall declines in ASMR and age-standardized DALY rates, the burden varies significantly across different regions and sociodemographic groups. Effective public health interventions, particularly in low- to middle-SDI regions, are essential to mitigate the impact of smoking-related ischemic stroke and improve global health outcomes.

## INTRODUCTION

Ischemic stroke remains a leading cause of morbidity and mortality worldwide, posing significant challenges to global health^1-3^. Characterized by the obstruction of blood flow to the brain, ischemic stroke results in severe neurological deficits and long-term disability^4,5^. Among numerous risk factors, active smoking has been identified as a major modifiable contributor to the incidence of ischemic stroke^6-8^. Understanding the burden of ischemic stroke attributable to active smoking is crucial for developing effective public health strategies and interventions.

Previous research has firmly established the detrimental effects of smoking on cardiovascular health, including a heightened risk of ischemic stroke^9-11^. Studies have explored various aspects of this relationship, such as the physiological mechanisms by which smoking induces vascular damage and the epidemiological trends in smoking-related stroke incidence^12-14^. However, comprehensive analyses covering long-term global, regional, and national perspectives, especially those exploring the period 1990–2021, remain rare.

The global burden of ischemic stroke attributable to active smoking remains a major challenge to quantify accurately. Existing studies rarely provide a comprehensive analysis that spans multiple decades and encompasses diverse geographical regions. The relationship between socio-economic development, as measured by the sociodemographic index (SDI), and the burden of smoking-related ischemic stroke is at present unclear. Addressing these gaps is essential to inform targeted public health interventions and policy decisions.

This study aims to fill these research gaps by analyzing the burden of ischemic stroke attributable to active smoking from 1990 to 2021, considering global, regional, and national perspectives. Utilizing data from the Global Burden of Disease (GBD) 2021 database, we investigate mortality and disabilityadjusted life years (DALYs) related to active smoking-induced ischemic stroke across various sociodemographic contexts. Our analysis includes the calculation of estimated annual percentage change (EAPC) and the establishment of joinpoint regression models to discern temporal trends and geographical variations. The findings of this study should provide valuable insights into the epidemiological patterns of smoking-related ischemic stroke and guide future public health strategies.

By providing a detailed examination of the temporal and geographical trends in the burden of ischemic stroke attributable to active smoking, this study underscores the critical need for targeted smoking cessation programs and stroke prevention strategies worldwide.

## METHODS

### Data acquisition

The GBD 2021 database (https://vizhub.healthdata.org/gbd-results/), created by the Institute for Health Metrics and Evaluation (IHME) at the University of Washington and supported by the Bill & Melinda Gates Foundation, provides the latest information on the distribution and burden of diseases and injuries across time, age, gender, location, and sociodemographic groups. Our analysis is based on a secondary study using the GBD database, which applies the comparative risk assessment (CRA) framework to estimate the burden of disease attributable to risk factors^15^. The CRA framework is based on the premise of determining how much of an outcome (ischemic stroke) can be attributed to controlling the exposure of a given risk factor (active smoking) to its theoretical minimum risk exposure level (TMREL)^15^. In this study, ischemic stroke (IS) is defined and classified according to the International Classification of Diseases (ICD) coding system. The GBD 2021 study uses a standardized framework to ensure consistency in how cerebrovascular diseases, including ischemic stroke, are reported globally. For fatal analysis, ischemic stroke is defined using ICD-10 codes: G45-G46.8, I63-I63.9, I65-I66.9, I67.2-I67.3, I67.5-I67.6, I69.3^16^. For non-fatal analysis, the same set of ICD-10 codes are used: I63-I63.9, I65-I66.9, I67.2-I67.3, I67.5-I67.6, I69.3^16^. We utilized this database to extract data on mortality and DALYs attributable to active smoking-related ischemic stroke for the globe, various SDI regions, different geographical areas, and 204 countries and territories^15,17^.

### Sociodemographic index

The sociodemographic index (SDI) is an important indicator of the development level of a country or region, ranging from 0 to 1, with higher values indicating higher socio-economic development. For this study, the GBD database categorizes different countries and regions into five SDI categories: low, low-middle, middle, high-middle, and high^18^.

### Statistical analysis

All data processing for this study was conducted using R Studio software (version 4.4.0). To analyze the time trend of the age-standardized mortality rate (ASMR) and age-standardized disability-adjusted life-year rate (ASDR) from 1990 to 2021, we used the estimated annual percentage change (EAPC) based on the annual age-standardized rate (ASR). EAPC assumes a linear relationship between ASR and time, modeled as y=α+βx+ε, where y represents log10 (ASR), x represents calendar year, and β represents the regression coefficient^19,20^. EAPC is calculated^19,20^ using the formula EAPC=100×(exp^β^-1). Unlike the 95% uncertainty interval (UI) used for other estimates, EAPC is accompanied by a 95% confidence interval (95% CI). ASR is considered to be on an increasing trend if both the lower limit of the EAPC and its 95% CI were greater than zero, and vice versa^19,20^.

To address the limitations of the estimated annual percentage change (EAPC) in capturing local variation, we calculated the standard errors of the global burden of disease (GBD) estimates by dividing the width of the 95% uncertainty interval (UI) by 3.92. By calculating these standard errors using the Delta method, we constructed 95% confidence intervals (CIs) and performed trend analyses. Joinpoint regression models were used to investigate temporal trends in active smoking in relation to ischemic stroke. Data analysis and visualization were performed using joinpoint software, with a log-linear model and a significance level set at α=0.05. The default method of modeling in joinpoint regression was the grid search method (GSM), supplemented by the Monte Carlo permutation method for model selection^19,20^. The joinpoint model can derive the annual percentage change (APC) over the study period, along with its 95% confidence interval (CI). A significant deviation of APC from zero indicates a trend classified as increasing (worsening) or decreasing (improving). Conversely, if the APC does not significantly differ from zero, the trend is considered stable or unchanged^19,20^. Statistical significance for all analyses was set at p<0.05, with two-tailed tests.

## RESULTS

### Global trends in ischemic stroke burden attributed to active smoking mortality

**Figure 1:**
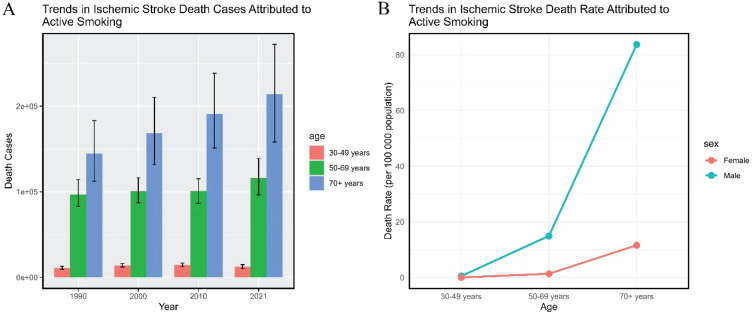
Global burden of ischemic stroke deaths attributable to active smoking: A) Trends in the number of deaths due to ischemic stroke caused by active smoking across all age groups from 1990 to 2021; B) Mortality rate of ischemic stroke caused by active smoking across all age groups in 2021

As shown in Table 1 and Figure 1, in 2021 there were 342674 (95% UI: 271782–420042) deaths globally due to ischemic stroke caused by active smoking, with an age-standardized mortality rate (ASMR) of 4.06 (95% UI: 3.18–4.98) and a population-attributable fraction (PAF) of 9.54% (95% UI: 7.76–11.63). Mortality rates were consistently higher in males than females across all age groups, with the highest rates observed in individuals aged ≥70 years.

**Table 1:** Deaths of ischemic stroke due to active smoking between 1990 and 2021, at global and regional level

*Location*	*1990*			*2021*			*1990–2021*	
	*Deaths* *Cases* *(95% UI)*	*PAF* *%* *(95% UI)*	*ASMR* *(95% UI)*	*Deaths* *Cases* *(95% UI)*	*PAF* *%* *(95% UI)*	*ASMR* *(95% UI)*	*Cases change* *%* *(95% UI)*	*EAPC* *(95% CI)*
Global	252737 208023–305598	10.91 9.06–13.07	7.06 5.71–8.66	342674 271782– 420042	9.54 7.76– 11.63	4.06 3.18–4.98	35.59 16.60–58.69	-1.94 -2.03 – -1.85
**Sociodemographic index**								
High	62208 50096–76748	10.45 8.42–12.70	5.52 4.46–6.81	34682 26474–44035	6.84 5.29–8.58	1.50 1.17–1.87	-44.25 -49.62 – -38.99	-4.59 -4.78 – -4.41
High-middle	91192 75375–108185	10.28 8.56–12.25	9.85 8.03–11.86	118032 92889–147430	10.25 8.27–12.56	5.95 4.67–7.45	29.43 9.08–53.46	-1.88 -2.15 – -1.62
Middle	66675 54330–83525	13.77 11.52–16.35	7.68 6.11–9.75	130726 101045–162483	11.15 9.03–13.65	5.28 4.04–6.64	96.06 54.30–147.13	-1.19 -1.26 – -1.12
Low-middle	26884 20821–34945	10.37 8.40–12.60	5.35 4.09–7.02	49692 38887–63033	8.54 6.89–10.36	3.93 3.03–5.00	84.84 58.29–119.38	-1.03 -1.08 – -0.99
Low	5393 4046–7540	6.18 4.94–7.64	3.00 2.22–4.09	9265 6994–12219	5.31 4.25–6.53	2.22 1.65–2.92	71.79 44.61–102.60	-1.07 -1.15 – -0.99
**Regions**								
Andean Latin America	292 227–362	5.30 4.22–6.49	1.57 1.21–1.96	389 280–524	3.97 3.07–5.10	0.69 0.49–0.92	33.19 5.55–68.90	-2.9 -3.2 – -2.6
Australasia	680 533–857	6.88 5.42–8.58	2.88 2.24–3.66	329 218–460	3.50 2.38–4.78	0.53 0.36–0.73	-51.65 -61.63 – -40.72	-5.63 -5.77 – -5.5
Caribbean	893 727–1110	7.46 6.02–9.23	3.66 2.94–4.58	1193 927–1507	5.98 4.75–7.49	2.20 1.71–2.78	33.53 13.65–56.94	-1.66 -1.77 – -1.54
Central Asia	2631 2191–3106	7.98 6.67–9.38	5.79 4.77–6.91	3773 3131–4471	8.41 7.09–9.85	5.03 4.11–6.01	43.38 26.86–63.79	-0.66 -1.03 – -0.29
Central Europe	18145 15111–21691	10.01 8.30–11.88	12.65 10.37–15.23	10286 8285–12651	6.42 5.19–7.76	4.44 3.60–5.44	-43.31 -48.70 – -37.19	-3.71 -3.92 – -3.5
Central Latin America	1834 1482–2222	7.55 6.11–9.04	2.57 2.02–3.18	1763 1346–2199	4.10 3.22–4.98	0.74 0.56–0.93	-3.90 -17.68–10.99	-4.39 -4.56 – -4.22
Central Sub-Saharan Africa	324 234–451	3.79 2.91–4.86	1.76 1.24–2.48	587 420–823	3.14 2.35–4.19	1.30 0.92–1.79	81.14 34.99–138.25	-1.05 -1.36 – -0.75
East Asia	76655 61072–98089	17.31 14.35–20.73	10.63 8.26–13.76	167795 124193–220928	13.95 11.11–17.18	8.15 5.99–10.69	118.90 58.82–199.95	-0.7 -0.87 – -0.52
Eastern Europe	29956 25339–34756	7.39 6.24–8.57	10.84 9.12–12.68	24351 19855–29421	7.40 6.11–8.94	6.81 5.57–8.21	-18.71 -29.26 – -8.93	-2.22 -2.93 – -1.51
Eastern Sub-Saharan Africa	1375 1007–1905	5.51 4.28–6.91	2.49 1.77–3.43	2250 1663–3068	4.34 3.37–5.53	1.69 1.25–2.31	63.62 22.45–118.75	-1.47 -1.56 – -1.39
High-income Asia Pacific	12565 10193–15366	11.90 9.70–14.39	6.81 5.47–8.42	6711 4808–8803	5.97 4.44–7.68	1.16 0.88–1.48	-46.59 -55.23 – -36.86	-6.28 -6.5 – -6.05
High-income North America	10607 8335–13354	9.64 7.66–11.86	2.86 2.26–3.59	7959 5906–10531	6.31 4.80–8.38	1.12 0.85–1.47	-24.97 -34.96 – -12.80	-3.69 -4.09 – -3.28
North Africa and Middle East	13012 10255–16343	9.93 8.24–11.94	9.12 7.08–11.50	21877 17389–27359	8.64 7.10–10.28	5.50 4.26–6.93	68.13 42.55–99.86	-1.76 -1.81 – -1.72
Oceania	68 49–93	8.41 6.83–10.29	2.72 1.95–3.77	127 90–176	6.90 5.56–8.47	1.95 1.36–2.68	87.78 45.58–146.94	-1.28 -1.36 – -1.19
South Asia	18902 13641–27234	10.92 8.52–13.57	4.19 3.00–5.96	35480 26731–50952	8.03 6.23–10.09	2.81 2.10–3.91	87.71 50.91–131.65	-1.46 -1.57 – -1.35
South-East Asia	16361 13061–20291	12.45 10.13–14.90	7.87 6.22–9.96	35887 25856–45712	10.49 8.26–12.93	6.21 4.54–8.01	119.35 71.47–175.94	-0.78 -0.92 – -0.64
Southern Latin America	1533 1212–1889	6.58 5.30–7.97	3.37 2.64–4.15	881 694–1118	4.49 3.54–5.56	0.98 0.78–1.24	-42.51 -50.32 – -32.96	-3.72 -3.82 – -3.62
Southern Sub-Saharan Africa	999 766–1254	10.20 8.36–12.20	4.32 3.21–5.55	1296 1025–1575	5.20 4.17–6.28	2.56 1.97–3.16	29.77 8.72–61.95	-1.75 -2.13 – -1.38
Tropical Latin America	8101 6676–9741	14.64 12.13–17.63	10.13 8.11–12.54	5438 4197–6861	7.61 5.93–9.55	2.18 1.67–2.77	-32.87 -41.38 – -23.73	-5.01 -5.25 – -4.77
Western Europe	36094 28269–44860	9.50 7.42–11.65	5.91 4.64–7.32	11290 8384–14736	5.25 3.94–6.71	1.00 0.77–1.28	-68.72 -72.04 – -65.74	-5.97 -6.18 – -5.77
Western Sub-Saharan Africa	1713 1297–2269	3.49 2.77–4.46	2.23 1.66–2.94	3012 2270–3981	3.24 2.53–4.10	1.72 1.28–2.25	75.79 37.94–130.34	-0.85 -1.01 – -0.7

ASMR: age-standardized mortality rate per 100000 population. EAPC: estimated annual percentage change. PAF: population attributable fraction. 95% UI: 95% uncertainty interval. 95% CI: 95% confidence interval.

From 1990 to 2021, the global number of deaths increased by 35.59%, with males aged ≥70 years experiencing the largest increase (64.27%). Conversely, female deaths decreased across all age groups, with the smallest decline in the ≥70 years age group (-5.79%). The global ASMR showed a decreasing trend, with an estimated annual percentage change (EAPC) of -1.94 (95% CI: -2.03 – -1.85). This decline was more pronounced in females (EAPC= -3.44; 95% CI: -3.60 – -3.28) compared to males (EAPC= -1.78; 95% CI: -1.86 – -1.71). The joinpoint regression model indicated that from 2018 to 2021, the global ASMR of ischemic stroke caused by active smoking continued to decline (APC= -1.19; 95% CI: -1.95 – -0.43). (Supplementary file: Table 1 and Figure 1).

*Disability-adjusted life years (DALYs)*

**Figure 2:**
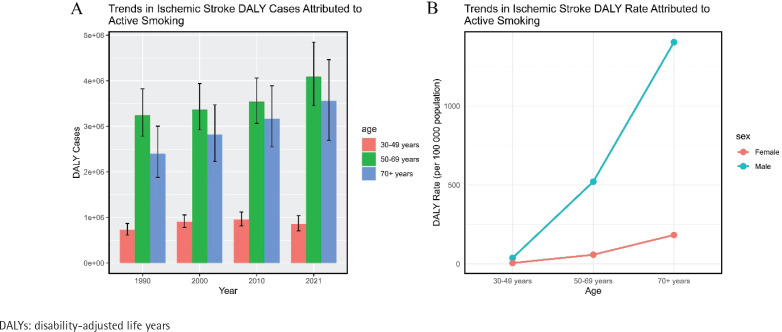
Global burden of ischemic stroke DALYs attributable to active smoking: A) Trends in the number of DALYs due to ischemic stroke caused by active smoking across all age groups from 1990 to 2021; B) DALYs rate of ischemic stroke caused by active smoking across all age groups in 2021

In 2021, the global number of DALYs due to ischemic stroke attributed to active smoking was 8510889 (95% UI: 7039201–10283725), with an age-standardized DALY rate of 98.29 (95% UI: 81.26–118.66). Similar to mortality rates, DALY rates were higher in males than females across all age groups, with the highest rates observed in individuals aged ≥70 years (Figure 2, and Supplementary file Table 2).

From 1990 to 2021, the number of DALYs increased by 33.55%, with the smallest decrease in males aged ≥70 years (-5.76%). Female DALYs decreased across all age groups, with the smallest decline in the ≥70 years age group (-5.28%). The global age-standardized DALY rate also showed a decreasing trend (EAPC= -1.78; 95% CI: -1.85 – -1.70). The decline was faster in females (EAPC= -3.07; 95% CI: -3.19 – -2.95) compared to males (EAPC= -1.64; 95% CI: -1.71 – -1.57). The joinpoint regression model demonstrated that from 2018 to 2021, the ASDR of ischemic stroke caused by active smoking worldwide exhibited a downward trend (APC= -1.18; 95% CI: -1.86 – -0.49) (Supplementary file: Table 1 and Figure 1).

### Regional trends in ischemic stroke burden attributed to active smoking

**Figure 3:**
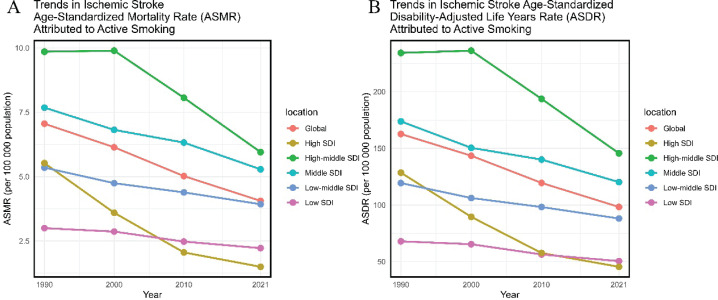
Disease burden of ischemic stroke due to active smoking at the SDI level from 1990 to 2021: A) Trends in ischemic stroke age-standardized mortality rate (ASMR) attributed to active smoking; B) Trends in ischemic stroke age-standardized disability-adijusted life years rate (ASDR) attributed to active smoking

Across various economic regions in 2021, the middle SDI region reported the highest number of deaths (130726; 95% UI: 101045–162483), PAF (11.15%; 95% UI: 9.03–13.65), and DALYs (3216256; 95% UI: 2560206–3910313). The high-middle SDI region had the highest ASMR (5.95; 95% UI: 4.67–7.45) and age-standardized DALY rate (145.65; 95% UI: 119.71–177.13). In contrast, the high SDI region recorded the lowest ASMR (1.50; 95% UI: 1.17–1.87) and ASDR (45.48; 95% UI: 37.29–55.66) (Figure 3 and Table 1, and Supplementary file Table 1).

From 1990 to 2021, the high SDI region experienced the largest decreases in deaths, ASMR, DALYs, and ASDR. Conversely, the middle SDI region saw the largest increases in deaths (96.06%) and DALYs (83.43%). The low-middle SDI region had the smallest declines in ASMR (EAPC= -1.03; 95% CI: -1.08 – -0.99) and ASDR (EAPC= -1.01; 95% CI: -1.04 – -0.98).

### Geographical trends in ischemic stroke burden attributed to active smoking mortality

In 2021, East Asia reported the highest number of deaths due to ischemic stroke attributed to active smoking (167795; 95% UI: 124193–220928), while Oceania had the lowest (127; 95% UI: 90–176). The highest PAF was also in East Asia (13.95%; 95% UI: 11.11–17.18), and the lowest was in Central Sub-Saharan Africa (3.14%; 95% UI: 2.35–4.19). East Asia had the highest ASMR (8.15; 95% UI: 5.99–10.69), whereas Australasia had the lowest (0.53; 95% UI: 0.36–0.73) (Supplementary file: Figure 2 and Table 1).

From 1990 to 2021, 12 regions experienced increases in the number of deaths due to active smoking-induced ischemic stroke, with the largest increase in South-East Asia (119.35%). Western Europe saw the largest decline (-68.72%). Notably, all 21 regions showed a decreasing trend in ASMR, with the smallest decline in Central Asia (EAPC= -0.66; 95% CI: -1.03 – -0.29) and the largest decline in the high-income Asia Pacific region (EAPC= -6.28; 95% CI: -6.50 – -6.05).

*DALYs*

In 2021, East Asia had the highest number of DALYs (3989934; 95% UI: 3103729–5123119), while Oceania had the lowest (4781; 95% UI: 3570–6265), consistent with the regions for mortality extremes. Eastern Europe had the highest ASDR (190.12; 95% UI: 161.37–225.54), while Andean Latin America had the lowest (15.95; 95% UI: 12.05–20.68) (Supplementary file: Figure 2 and Table 1).

From 1990 to 2021, 12 regions saw increases in DALYs, with 9 regions experiencing increases above the global average. South-East Asia had the largest increase (120.51%). All 21 regions exhibited a decreasing trend in ASDR, with 11 regions showing declines below the global average. South-East Asia had the smallest decline (EAPC= -0.63; 95% CI: -0.72 – -0.54).

### National trends in ischemic stroke burden attributed to active smoking mortality

**Figure 4:**
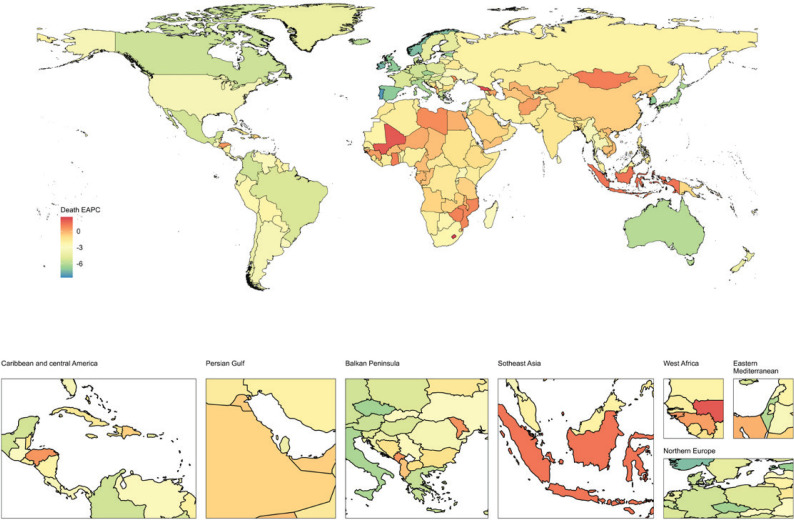
National burden of ischemic stroke attributable to active smoking across 204 countries and territories, represented by the estimated annual percentage change (EAPC) in age-standardized mortality rate (ASMR), 1990–2021

In 2021, China had the highest number of deaths due to smoking-related ischemic stroke (165387; 95% UI: 122395–218443). Kiribati had the highest PAF (19.24%; 95% UI: 16.14–22.83), while Ethiopia had the lowest (1.63%; 95% UI: 1.16–2.23). North Macedonia had the highest ASMR (17.35; 95% UI: 11.98–24.40), and Singapore had the lowest (0.24; 95% UI: 0.18–0.32) (Figure 4, and Supplementary file: Table 3 and Figure 3).

From 1990 to 2021, 127 countries and regions saw an increase in the number of deaths, with Djibouti experiencing the largest increase (338.87%). ASMR increased in 21 countries and regions, with Lesotho showing the highest increase (EAPC=2.61; 95% CI: 2.23–2.98). Among the 183 countries and regions with a decline in ASMR, 78 showed decreases below the global average. Singapore had the largest decrease (EAPC= -8.56, 95% CI: -8.92 – -8.21).

*DALYs*

**Figure 5:**
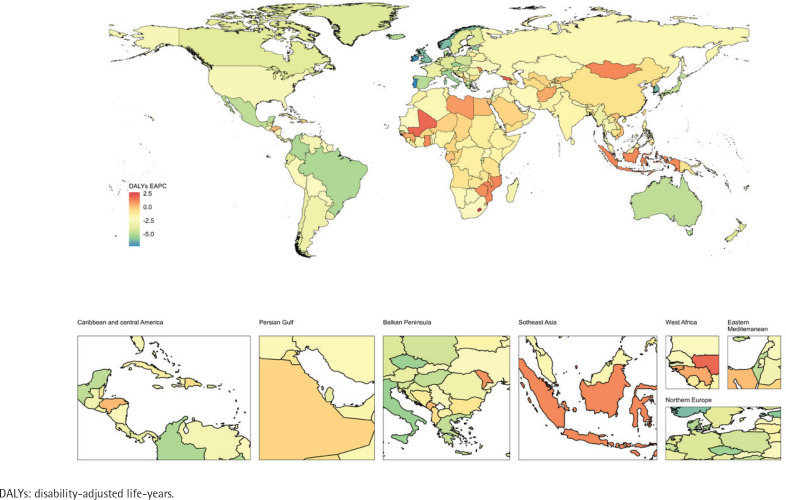
National burden of ischemic stroke attributable to active smoking across 204 countries and territories, represented by the estimated annual percentage change (EAPC) in age-standardized dalys rate (ASDR), 1990–2021

In 1990, China had the highest number of DALYs due to smoking-related ischemic stroke (3916454; 95% UI: 3034617–5040372). North Macedonia had the highest ASDR (323.33; 95% UI: 240.27–425.26), while Ethiopia had the lowest (9.55; 95% UI: 6.54–14.10) (Figure 5, and Supplementary file: Table 4 and Figure 3).

From 1990 to 2021, 129 countries and regions saw an increase in DALYs, with Qatar experiencing the largest increase (336.49%). ASDR increased in 21 countries and regions, with Lesotho showing the highest increase (EAPC=2.7; 95% CI: 2.36–3.04). Among the 183 countries with a decline, 82 showed decreases below the global average. Portugal had the largest decrease (EAPC= -7.24; 95% CI: -7.51 – -6.96).

## DISCUSSION

In 2021, active smoking led to 6175019 deaths and 165080664 disability-adjusted life years (DALYs) globally, with the population attributable fraction (PAF) of current smokers being 9.10%. This study provides a comprehensive secondary analysis of the GBD 2021 database, revealing global, regional, and national trends in ischemic stroke attributable to active smoking over the past 32 years. We observed a significant increase in the global burden of ischemic stroke, measured by deaths and DALYs, from 1990 to 2021. Despite the increase in absolute numbers, age-standardized mortality rates (ASMR) and agestandardized DALY rates (ASDR) have shown a declining trend, indicating potential improvements in stroke prevention and management. However, this decline is not uniform across different regions and sociodemographic backgrounds.

Our findings show a 35.59% increase in global deaths due to ischemic stroke attributable to active smoking, with the highest increase observed in males, particularly those aged ≥70 years. This aligns with previous studies. Barengo et al.^21^ emphasized the need for smoking cessation counseling targeting older adults, given the significant risk difference between non-smokers and current smokers in this age group. Pan et al.^22^ also reported an overall increased stroke risk among smokers, with higher risks in males and lower risks in females compared to non-smokers. Additionally, the observed decline in global ASMR (EAPC= -1.94) and ASDR (EAPC= -1.78) is consistent with trends reported for the burden of ischemic heart disease attributable to active smoking, where improvements in healthcare and smoking cessation programs were cited as contributing factors^23^. The more pronounced decline in females (EAPC: ASMR= -3.44, ASDR= -3.07) suggests gender differences in health behaviors and access to healthcare services.

Our regional analysis underscores significant disparities in the burden of ischemic stroke attributable to active smoking. The middle SDI region reported the highest number of deaths and DALYs, while the high SDI region reported the lowest. This contrast highlights the impact of socio-economic development on health outcomes, as higher SDI regions likely benefit from better healthcare infrastructure, public health policies, and smoking cessation programs^24-26^. The substantial increases in deaths (96.06%) and DALYs (83.43%) in the middle SDI region raise concerns about the adequacy of current public health interventions in these areas. Although the low SDI region exhibits a lower burden, this may be influenced by limited healthcare access, reducing the number of detectable ischemic stroke cases^27^.

Geographically, East Asia had the highest burden of ischemic stroke attributable to active smoking in 2021, with the greatest number of deaths and DALYs. The high population density and smoking rates in this region contribute to its elevated burden^28-30^. In contrast, regions such as Western Europe and the high-income Asia Pacific region showed significant declines in ASMR and ASDR, reflecting effective public health strategies and improvements in healthcare in high-income areas. The smallest decline in ASMR in Central Asia and the largest increase in deaths in South-East Asia highlight the need for region-specific interventions to combat smoking-related strokes.

At the national level, China had the highest number of deaths and DALYs due to smoking-related ischemic stroke, reflecting its high smoking rates and large population^31,32^. North Macedonia’s significant increase in ASMR and ASDR indicates emerging public health challenges amid socio-economic transitions. Tobacco production is a critical economic and social factor in North Macedonia, providing substantial income and livelihood for the rural population due to its high-quality raw materials^33^. Nearly 700000 smokers in North Macedonia consume an average of 11 tons of tobacco annually, and the number of smokers has been rising over the past 15 years^34^. Therefore, new national smoking cessation programs are necessary, as current programs frequently fail and require revisions^35^. The sharp declines in ASMR and ASDR in countries like Singapore and Portugal reflect successful public health policies, including proactive smoking cessation programs and improved stroke care.

### Limitations

Our study has some limitations. Our study was based on a secondary analysis of a public database, so we could not analyze the effects of each subtype of ischemic stroke at the individual level. Our study relies on data from the GBD database, which inherently presents limitations related to residual confounding factors. Furthermore, the GBD database does not offer comprehensive regional data for all countries. This limitation is particularly relevant for countries, such as China, where national-level data are available, but regional-level data may be lacking. In such cases, comparisons must often be made between national data and that of relevant public health authorities or CDCs. Additionally, modeling the effects of tobacco use on stroke incidence is further complicated by differences in smoking cessation policies, making accurate predictions more challenging in real-world scenarios.

## CONCLUSIONS

This study underscores the significant global burden of ischemic stroke attributable to active smoking and the critical need for targeted smoking cessation programs and stroke prevention strategies. Our findings contribute to the broader understanding of the public health impact of smoking and offer a foundation for future research and policy development. Continued efforts to reduce smoking prevalence, particularly in low- to middle-SDI regions, are essential to mitigate the global burden of ischemic stroke and improve population health outcomes.

## Supplementary Material





## Data Availability

The data supporting this research are available from the authors on reasonable request.
