# Supplementary material for: Global, regional, and national burden of ischemic stroke attributable to active smoking, 1990–2021
Source: Tob Induc Dis. 2024 Nov 8;22:10.18332/tid/194697. doi: 10.18332/tid/194697 (PMC11541932; doi:10.18332/tid/194697)
Supplement: Supplementary file 1 [file TID-22-176-s1.pdf]

**Supplementary Figure Legend:**

**Supplementary Figure 1. The APC of death, and DALYs of Ischemic Stroke due to active smoking between 1990 and 2021 at the global level.**

**Supplementary Figure 2. ASMR, and ASDR for ischemic stroke due to active smoking at the Regional level From 1990 to 2021.**

**Supplementary Figure 3. The national burden of ischemic stroke due to active smoking in 204 countries and territories.**

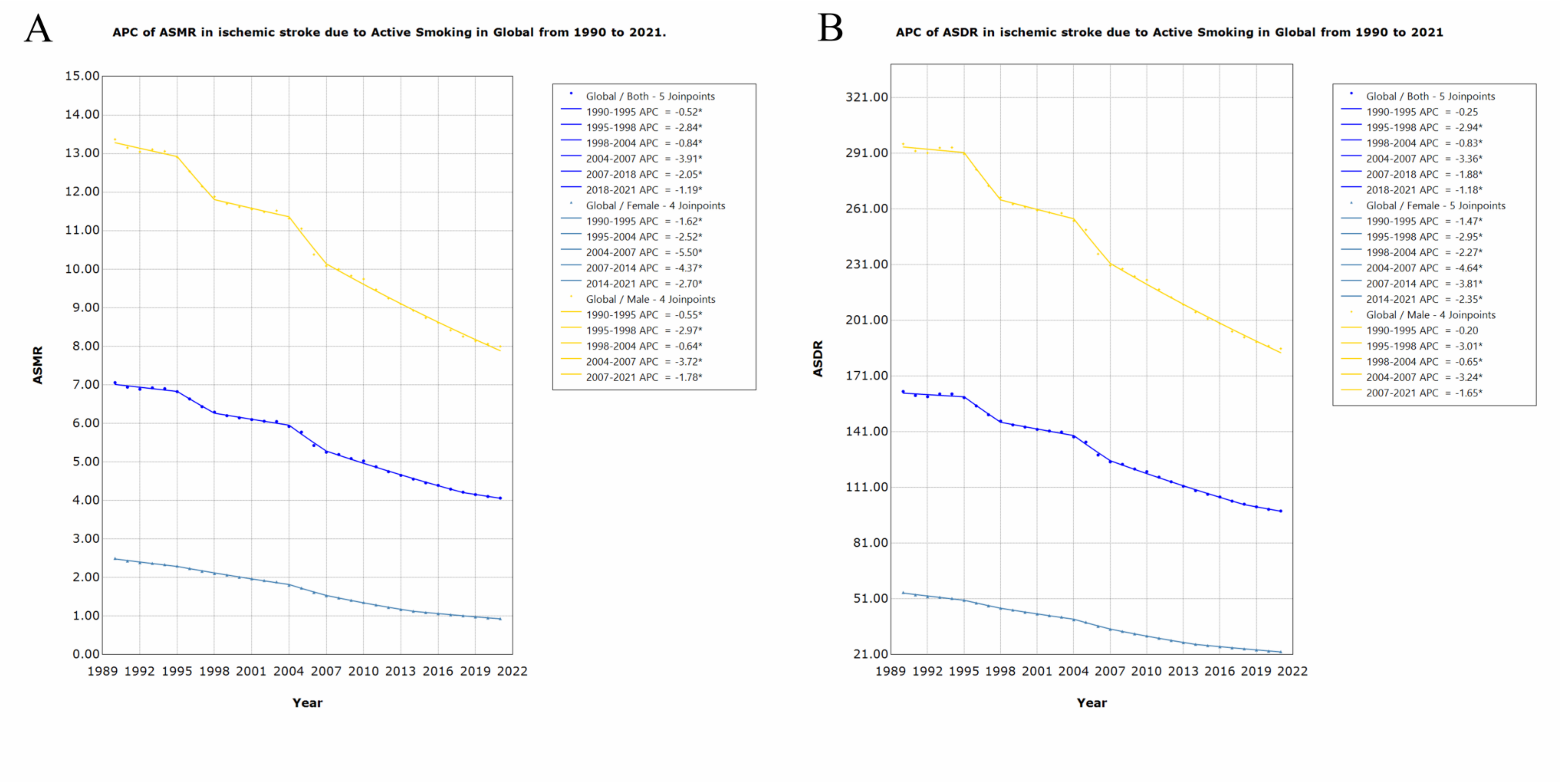
A
APC of ASMR in ischemic stroke due to Active Smoking in Global from 1990 to 2021.
ASMR
Year
Global / Both - 5 Joinpoints
1990-1995 APC = -0.52*
1995-1998 APC = -2.84*
1998-2004 APC = -0.84*
2004-2007 APC = -3.91*
2007-2018 APC = -2.05*
2018-2021 APC = -1.19*
Global / Female - 4 Joinpoints
1990-1995 APC = -1.62*
1995-2004 APC = -2.52*
2004-2007 APC = -5.50*
2007-2014 APC = -4.37*
2014-2021 APC = -2.70*
Global / Male - 4 Joinpoints
1990-1995 APC = -0.55*
1995-1998 APC = -2.97*
1998-2004 APC = -0.64*
2004-2007 APC = -3.72*
2007-2021 APC = -1.78*
B
APC of ASDR in ischemic stroke due to Active Smoking in Global from 1990 to 2021
ASDR
Year
Global / Both - 5 Joinpoints
1990-1995 APC = -0.25
1995-1998 APC = -2.94*
1998-2004 APC = -0.83*
2004-2007 APC = -3.36*
2007-2018 APC = -1.88*
2018-2021 APC = -1.18*
Global / Female - 5 Joinpoints
1990-1995 APC = -1.47*
1995-1998 APC = -2.95*
1998-2004 APC = -2.27*
2004-2007 APC = -4.64*
2007-2014 APC = -3.81*
2014-2021 APC = -2.35*
Global / Male - 4 Joinpoints
1990-1995 APC = -0.20
1995-1998 APC = -3.01*
1998-2004 APC = -0.65*
2004-2007 APC = -3.24*
2007-2021 APC = -1.65*

**Supplementary Figure 1. The APC of death, and DALYs of Ischemic Stroke due to active smoking between 1990 and 2021 at the global level. (A) APC of ASMR in ischemic stroke due to Active Smoking in Global from 1990 to 2021. (B) APC of ASDR in ischemic stroke due to Active Smoking in Global from 1990 to 2021.**

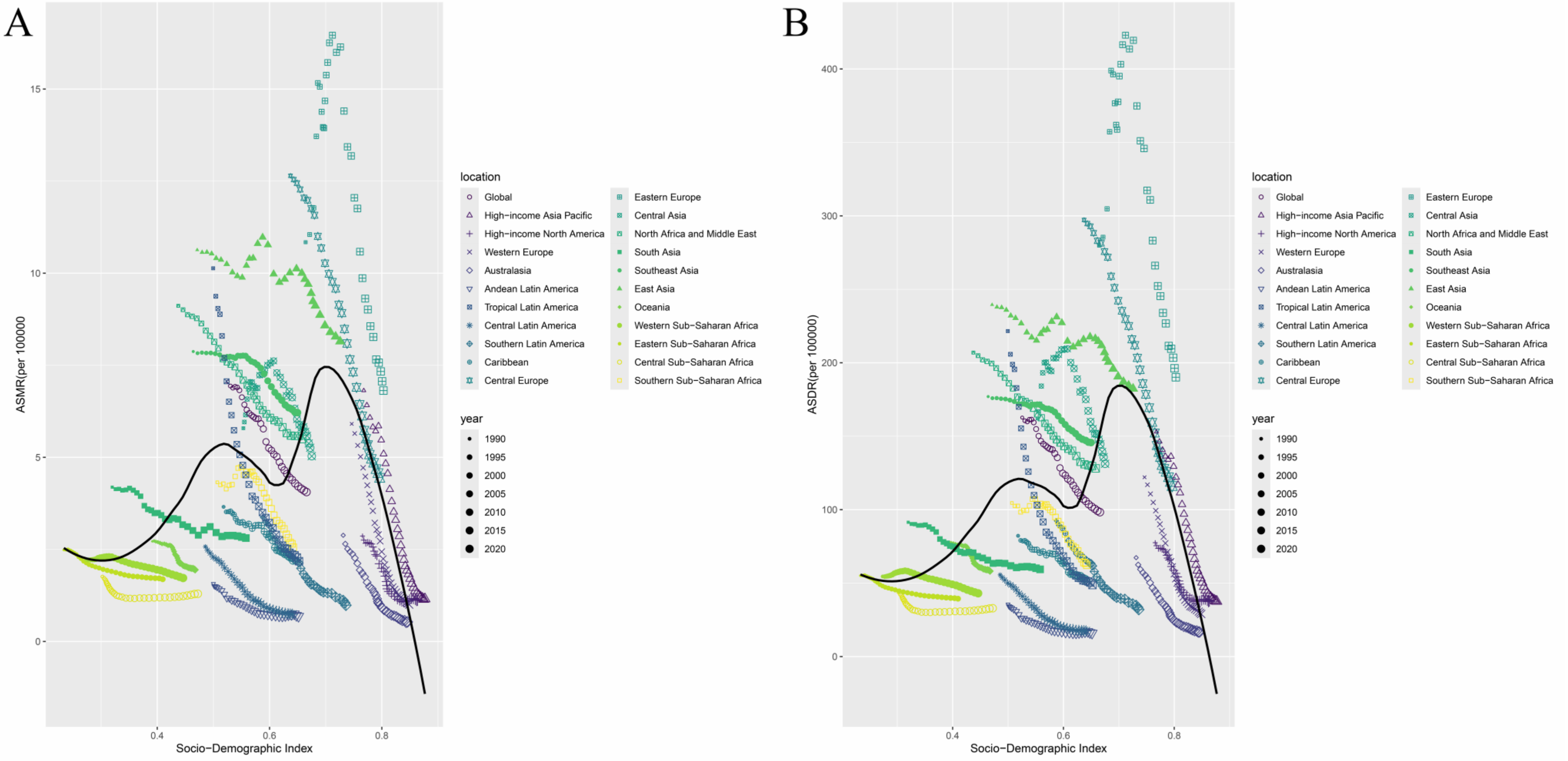
A
ASMR(per 100000
15
10
5
0
0.4
0.6
0.8
Socio−Demographic Index
B
ASDR(per 100000)
400
300
200
100
0
0.4
0.6
0.8
Socio−Demographic Index
location
Global
High−income Asia Pacific
High−income North America
Western Europe
Australasia
Andean Latin America
Tropical Latin America
Central Latin America
Southern Latin America
Caribbean
Central Europe
Eastern Europe
Central Asia
North Africa and Middle East
South Asia
Southeast Asia
East Asia
Oceania
Western Sub−Saharan Africa
Eastern Sub−Saharan Africa
Central Sub−Saharan Africa
Southern Sub−Saharan Africa
year
1990
1995
2000
2005
2010
2015
2020

**Supplementary Figure 2. ASMR, and ASDR for ischemic stroke due to active smoking at the Regional level From 1990 to 2021. (A) ASMR. (B) ASDR. ASMR = Age-standardized mortality rate (per 100000 population). ASDR = Age-standardized DALYs rate (per 100000 population).**

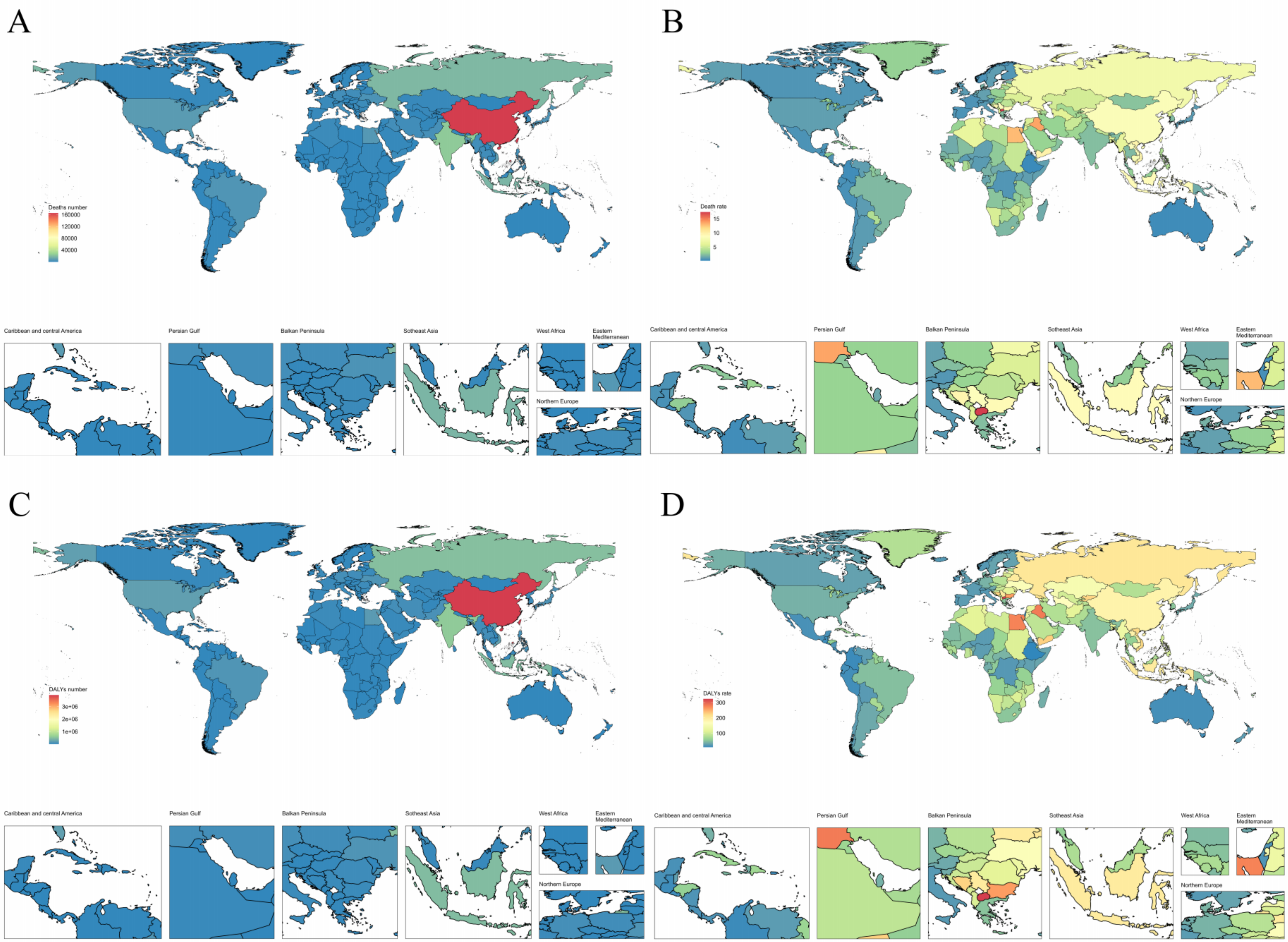
A
Deaths number
160000
120000
80000
40000
Caribbean and central America
Persian Gulf
Balkan Peninsula
Sotheast Asia
West Africa
Eastern Mediterranean
Northern Europe
B
Death rate
15
10
5
Caribbean and central America
Persian Gulf
Balkan Peninsula
Sotheast Asia
West Africa
Eastern Mediterranean
Northern Europe
C
DALYs number
3e+06
2e+06
1e+06
Caribbean and central America
Persian Gulf
Balkan Peninsula
Sotheast Asia
West Africa
Eastern Mediterranean
Northern Europe
D
DALYs rate
300
200
100
Caribbean and central America
Persian Gulf
Balkan Peninsula
Sotheast Asia
West Africa
Eastern Mediterranean
Northern Europe

**Supplementary Figure 3. The national burden of ischemic stroke due to active smoking in 204 countries and territories. (A) Disease burden of deaths cases. (B) Disease burden of deaths rate. (C) Disease burden of DALYs cases. (D) Disease burden of DALYs rate.**

© 2024 Pang M. et al.
