# Supplementary material for: Global, regional, and national burden of ischemic stroke attributable to active smoking, 1990–2021
Source: Tob Induc Dis. 2024 Nov 8;22:10.18332/tid/194697. doi: 10.18332/tid/194697 (PMC11541932; doi:10.18332/tid/194697)
Supplement: Supplementary file 2 [file TID-22-176-s2.pdf]

Supplementary Table 1. The APC of death, and DALYs of Ischemic Stroke due to active smoking between 1990 and 2021 at the global level. APC = Annual percent change. DALYs = Disability-Adjusted Life Years. **95%CI = 95% Confidence Interval.**

Measure	location	sex	Segment Start	Segment End	APC (95% CI)	Test Statistic	P-Value
Deaths	Global	Both	1990	1995	-0.52 (-0.85 to -0.20)	-3.422	0.004
Deaths	Global	Both	1995	1998	-2.84 (-4.20 to -1.46)	-4.348	0.001
Deaths	Global	Both	1998	2004	-0.85 (-1.15 to -0.54)	-5.797	<0.001
Deaths	Global	Both	2004	2007	-3.91 (-5.27 to -2.53)	-5.973	<0.001
Deaths	Global	Both	2007	2018	-2.05 (-2.16 to -1.94)	-40.184	<0.001
Deaths	Global	Both	2018	2021	-1.19 (-1.95 to -0.43)	-3.316	0.005
Deaths	Global	Female	1990	1995	-1.62 (-1.88 to -1.35)	-12.77	<0.001
Deaths	Global	Female	1995	2004	-2.52 (-2.65 to -2.39)	-39.777	<0.001
Deaths	Global	Female	2004	2007	-5.50 (-6.67 to -4.31)	-9.503	<0.001
Deaths	Global	Female	2007	2014	-4.37 (-4.58 to -4.17)	-44.014	<0.001
Deaths	Global	Female	2014	2021	-2.70 (-2.88 to -2.53)	-31.86	<0.001
Deaths	Global	Male	1990	1995	-0.55 (-0.97 to -0.12)	-2.699	0.015
Deaths	Global	Male	1995	1998	-2.97 (-4.75 to -1.17)	-3.435	0.003
Deaths	Global	Male	1998	2004	-0.64 (-1.04 to -0.24)	-3.347	0.004
Deaths	Global	Male	2004	2007	-3.72 (-5.46 to -1.94)	-4.35	<0.001
Deaths	Global	Male	2007	2021	-1.78 (-1.87 to -1.69)	-42.017	<0.001
DALYs	Global	Both	1990	1995	-0.25 (-0.53 to 0.04)	-1.859	0.083
DALYs	Global	Both	1995	1998	-2.94 (-4.13 to -1.74)	-5.168	<0.001
DALYs	Global	Both	1998	2004	-0.84 (-1.10 to -0.57)	-6.639	<0.001
DALYs	Global	Both	2004	2007	-3.36 (-4.53 to -2.18)	-6.005	<0.001
DALYs	Global	Both	2007	2018	-1.88 (-1.98 to -1.79)	-42.498	<0.001
DALYs	Global	Both	2018	2021	-1.18 (-1.86 to -0.49)	-3.641	0.002
DALYs	Global	Female	1990	1995	-1.47 (-1.70 to -1.24)	-13.561	<0.001
DALYs	Global	Female	1995	1998	-2.95 (-3.96 to -1.93)	-6.08	<0.001
DALYs	Global	Female	1998	2004	-2.27 (-2.50 to -2.04)	-20.804	<0.001
DALYs	Global	Female	2004	2007	-4.64 (-5.66 to -3.60)	-9.388	<0.001
DALYs	Global	Female	2007	2014	-3.81 (-3.98 to -3.63)	-45.79	<0.001
DALYs	Global	Female	2014	2021	-2.35 (-2.51 to -2.20)	-31.882	<0.001
DALYs	Global	Male	1990	1995	-0.21 (-0.57 to 0.16)	-1.178	0.254
DALYs	Global	Male	1995	1998	-3.01 (-4.48 to -1.51)	-4.192	0.001
DALYs	Global	Male	1998	2004	-0.65 (-0.98 to -0.31)	-4.031	0.001
DALYs	Global	Male	2004	2007	-3.24 (-4.70 to -1.75)	-4.536	<0.001
DALYs	Global	Male	2007	2021	-1.65 (-1.72 to -1.57)	-45.665	<0.001

Supplementary Table 2. DALYs of Ischemic Stroke due to active smoking between 1990 and 2021 at the global and regional levels. DALYs = Disability-Adjusted Life Years. ASDR = Age-standardized DALYs rate (per 100000 population). EAPC = estimated annual percentage change. **95%UI = 95% Uncertainty Interval. 95%CI = 95% Confidence Interval.**

Location	1990		2021		1990-2021	
	DALYs Cases (95%UI)	ASDR (95%UI)	DALYs Cases (95%UI)	ASDR (95%UI)	Cases change, % (95%UI)	EAPC (95%CI)
**Global**	6372606 (5375527 to 7533255)	162.68 (136.07 to 193.54)	8510889 (7039201 to 10283725)	98.29 (81.26 to 118.66)	33.55 (16.57 to 53.48)	-1.78 (-1.85 to -1.7)
**Socio-demographic index**						
High	1423422 (1199123 to 1694562)	128.56 (108.48 to 152.86)	897930 (729645 to 1108880)	45.48 (37.29 to 55.66)	-36.92 (-41.75 to -31.83)	-3.65 (-3.81 to -3.49)
High-middle	2336954 (1990233 to 2740359)	234.26 (196.91 to 275.89)	2890289 (2370698 to 3517453)	145.65 (119.71 to 177.13)	23.68 (6.04 to 44.21)	-1.82 (-2.06 to -1.57)
Middle	1753444 (1459783 to 2140083)	173.82 (143.17 to 213.71)	3216256 (2560206 to 3910313)	120.23 (95.73 to 147.18)	83.43 (45.41 to 127.30)	-1.15 (-1.21 to -1.09)
Low-middle	701418 (553703 to 900919)	119.38 (93.46 to 154.51)	1247267 (995320 to 1566962)	88.11 (70.42 to 111.11)	77.82 (53.21 to 107.08)	-1.01 (-1.04 to -0.98)
Low	147940 (115214 to 198089)	67.95 (52.05 to 91.84)	252216 (197524 to 325059)	50.49 (39.45 to 65.58)	70.49 (45.67 to 98.70)	-1.12 (-1.2 to -1.04)
**Regions**						
Andean Latin America	7261 (5867 to 8816)	35.60 (28.54 to 43.36)	9441 (7131 to 12208)	15.95 (12.05 to 20.68)	30.03 (5.18 to 59.66)	-2.77 (-3.06 to -2.47)
Australasia	16026 (13081 to 19562)	67.31 (54.97 to 82.36)	8534 (6605 to 11233)	16.71 (13.13 to 21.80)	-46.74 (-53.67 to -39.25)	-4.64 (-4.82 to -4.46)
Caribbean	21260 (17752 to 25358)	82.20 (68.43 to 98.76)	27471 (22143 to 33666)	50.95 (41.05 to 62.45)	29.22 (11.12 to 50.56)	-1.54 (-1.63 to -1.44)

Location	1990		2021		1990-2021	
	**DALYs Cases (95%UI)**	**ASDR (95%UI)**	**DALYs Cases (95%UI)**	**ASDR (95%UI)**	**Cases change, % (95%UI)**	**EAPC (95%CI)**
Central Asia	78975 (67049 to 91608)	162.07 (137.13 to 188.82)	111574 (93590 to 131248)	131.22 (109.24 to 154.55)	41.28 (26.92 to 59.75)	-1 (-1.4 to -0.6)
Central Europe	444750 (380391 to 519429)	297.21 (254.31 to 347.90)	247811 (204611 to 296987)	116.07 (96.33 to 138.32)	-44.28 (-48.84 to -38.82)	-3.34 (-3.5 to -3.17)
Central Latin America	45492 (38521 to 53357)	55.60 (46.19 to 66.12)	43538 (35317 to 53298)	17.41 (14.10 to 21.38)	-4.29 (-15.62 to 7.84)	-4.13 (-4.33 to -3.93)
Central Sub-Saharan Africa	9860 (7333 to 13374)	43.68 (32.31 to 58.97)	18603 (13721 to 25373)	32.88 (24.04 to 44.97)	88.68 (48.04 to 139.84)	-0.95 (-1.23 to -0.67)
East Asia	2027884 (1641091 to 2554290)	239.51 (192.11 to 300.36)	3989934 (3103729 to 5123119)	182.35 (141.22 to 232.52)	96.75 (44.79 to 162.14)	-0.72 (-0.84 to -0.6)
Eastern Europe	795571 (684937 to 914751)	279.76 (241.84 to 321.64)	654184 (552984 to 777602)	190.12 (161.37 to 225.54)	-17.77 (-26.89 to -9.10)	-1.97 (-2.63 to -1.3)
Eastern Sub-Saharan Africa	37961 (29590 to 51029)	55.07 (41.56 to 73.04)	64417 (49212 to 83905)	39.38 (30.04 to 52.36)	69.69 (30.94 to 119.03)	-1.28 (-1.36 to -1.2)
High-income Asia Pacific	303113 (256568 to 359739)	152.97 (128.42 to 182.21)	162138 (126652 to 204854)	37.57 (30.03 to 46.96)	-46.51 (-52.65 to -39.28)	-5.02 (-5.21 to -4.82)
High-income North America	272391 (223944 to 334773)	77.52 (64.07 to 95.11)	232558 (181663 to 295147)	37.83 (29.96 to 47.46)	-14.62 (-22.21 to -6.85)	-2.79 (-3.05 to -2.53)
North Africa and Middle East	347460 (280978 to 429374)	206.92 (165.38 to 255.31)	598395 (486046 to 723483)	128.08 (103.62 to 157.57)	72.22 (47.08 to 103.07)	-1.65 (-1.68 to -1.61)
Oceania	2443 (1849 to 3189)	77.48 (58.52 to 101.62)	4781 (3570 to 6265)	58.16 (42.88 to 76.12)	95.71 (57.35 to 147.46)	-1.11 (-1.19 to -1.03)
South Asia	493958 (365267 to 704604)	91.41 (66.87 to 128.69)	844162 (646420 to 1216533)	59.36 (45.72 to 85.12)	70.90 (41.28 to 104.98)	-1.56 (-1.65 to -1.47)

Location	1990		2021		1990-2021	
	**DALYs Cases (95%UI)**	**ASDR (95%UI)**	**DALYs Cases (95%UI)**	**ASDR (95%UI)**	**Cases change, % (95%UI)**	**EAPC (95%CI)**
Southeast Asia	432316 (350019 to 527644)	176.97 (143.21 to 219.32)	953297 (700747 to 1191618)	145.68 (107.80 to 182.69)	120.51 (74.28 to 171.78)	-0.63 (-0.72 to -0.54)
Southern Latin America	43194 (35663 to 52010)	92.31 (75.99 to 111.72)	27262 (22061 to 33530)	31.98 (25.92 to 39.06)	-36.88 (-44.05 to -28.76)	-3.34 (-3.42 to -3.26)
Southern Sub-Saharan Africa	28087 (22581 to 34078)	104.53 (82.03 to 127.79)	36201 (29865 to 43002)	62.16 (50.71 to 74.83)	28.89 (11.42 to 51.51)	-1.71 (-2 to -1.42)
Tropical Latin America	200259 (170594 to 231988)	221.57 (185.92 to 261.78)	125203 (101494 to 154756)	48.69 (39.26 to 60.38)	-37.48 (-44.20 to -30.08)	-5.08 (-5.36 to -4.8)
Western Europe	715522 (585400 to 864044)	122.06 (100.89 to 145.91)	260980 (207792 to 327098)	28.98 (23.38 to 35.82)	-63.53 (-66.49 to -60.74)	-4.82 (-5.02 to -4.61)
Western Sub-Saharan Africa	48825 (38064 to 63830)	54.49 (42.49 to 71.26)	90405 (69616 to 118795)	43.05 (33.15 to 55.65)	85.16 (46.98 to 137.16)	-0.81 (-1 to -0.62)

Supplementary Table 3. Deaths of Ischemic Stroke due to active smoking at the national level. ASMR = Age-standardized mortality rate (per 100000 population). EAPC = estimated annual percentage change. PAF = population attributable fraction. **95%UI = 95% Uncertainty Interval. 95%CI = 95% Confidence Interval.**

Location	1990			2021			1990-2021	
	Deaths Cases (95%UI)	PAF, % (95%UI)	ASMR (95%UI)	Deaths Cases (95%UI)	PAF, % (95%UI)	ASMR (95%UI)	Cases change, % (95%UI)	EAPC (95%CI)
Afghanistan	279 (170 to 438)	4.43 (3.29 to 6.08)	4.61 (2.78 to 7.40)	420 (282 to 643)	5.55 (4.26 to 7.07)	4.92 (3.28 to 7.43)	50.44 (1.07 to 120.76)	0.32 (-0.09 to 0.73)
Albania	126 (91 to 163)	14.91 (11.48 to 18.27)	7.59 (5.46 to 9.94)	243 (160 to 341)	13.41 (10.03 to 17.17)	5.74 (3.78 to 8.02)	92.79 (26.05 to 182.43)	-0.49 (-0.81 to -0.18)
Algeria	869 (605 to 1181)	11.06 (8.34 to 14.45)	11.51 (7.76 to 16.15)	1575 (1089 to 2274)	8.25 (6.12 to 10.91)	6.44 (4.23 to 9.29)	81.27 (32.04 to 148.32)	-1.81 (-1.95 to -1.67)
American Samoa	1 (0 to 1)	9.01 (7.04 to 11.08)	2.92 (2.13 to 3.85)	1 (1 to 1)	6.97 (5.44 to 8.84)	1.96 (1.43 to 2.66)	53.08 (13.08 to 109.91)	-1.73 (-1.96 to -1.51)
Andorra	1 (1 to 2)	9.55 (7.17 to 12.50)	2.33 (1.55 to 3.36)	1 (1 to 2)	5.33 (3.65 to 7.41)	0.80 (0.50 to 1.19)	13.62 (-32.95 to 74.03)	-3.33 (-3.64 to -3.03)
Angola	96 (64 to 140)	6.43 (4.94 to 8.30)	3.15 (2.07 to 4.46)	233 (160 to 322)	5.47 (4.15 to 6.89)	2.51 (1.72 to 3.54)	142.62 (59.98 to 257.65)	-0.86 (-1.1 to -0.61)
Antigua and Barbuda	1 (1 to 2)	3.79 (2.77 to 5.08)	2.28 (1.71 to 3.01)	1 (1 to 2)	4.04 (3.02 to 5.34)	1.32 (0.97 to 1.80)	-1.61 (-18.53 to 22.56)	-2.09 (-2.41 to -1.76)
Argentina	1094 (844 to 1350)	7.03 (5.56 to 8.72)	3.44 (2.62 to 4.28)	595 (464 to 752)	5.23 (4.08 to 6.55)	1.03 (0.81 to 1.30)	-45.63 (-54.86 to -33.94)	-3.54 (-3.69 to -3.38)
Armenia	181 (144 to 217)	10.99 (8.94 to 13.07)	7.35 (5.75 to 9.00)	251 (198 to 304)	11.44 (9.41 to 13.70)	5.72 (4.52 to 6.92)	39.11 (17.42 to 69.41)	-1.71 (-2.06 to -1.36)
Australia	534 (417 to 677)	6.53 (5.13 to 8.20)	2.72 (2.10 to 3.46)	232 (153 to 335)	3.07 (2.02 to 4.28)	0.44 (0.30 to 0.62)	-56.56 (-67.03 to -44.03)	-6.04 (-6.16 to -5.92)
Austria	525 (404 to 673)	6.19 (4.75 to 7.88)	4.22 (3.28 to 5.33)	178 (127 to 242)	5.61 (4.01 to 7.39)	0.85 (0.62 to 1.14)	-66.06 (-72.81 to -58.62)	-5.51 (-5.77 to -5.25)

Location	1990			2021			1990-2021	
	Deaths Cases (95%UI)	PAF, % (95%UI)	ASMR (95%UI)	Deaths Cases (95%UI)	PAF, % (95%UI)	ASMR (95%UI)	Cases change, % (95%UI)	EAPC (95%CI)
Azerbaijan	192 (135 to 263)	10.55 (8.26 to 13.54)	4.07 (2.88 to 5.56)	319 (215 to 445)	11.10 (8.10 to 14.66)	3.76 (2.56 to 5.27)	65.64 (9.88 to 155.49)	0.24 (-0.18 to 0.67)
Bahamas	2 (2 to 3)	4.39 (3.38 to 5.64)	1.61 (1.17 to 2.14)	4 (3 to 5)	4.07 (3.00 to 5.52)	1.04 (0.71 to 1.46)	64.94 (16.63 to 126.56)	-1.35 (-1.5 to -1.19)
Bahrain	9 (7 to 12)	11.08 (8.83 to 13.97)	7.78 (5.56 to 10.33)	20 (15 to 26)	9.62 (7.74 to 12.04)	3.80 (2.69 to 5.29)	122.84 (67.11 to 189.33)	-2.88 (-3.3 to -2.46)
Bangladesh	3136 (2060 to 4768)	10.85 (8.31 to 14.07)	7.88 (5.19 to 11.69)	7301 (4812 to 10789)	9.23 (6.85 to 11.80)	6.21 (4.11 to 9.24)	132.83 (71.08 to 231.03)	-0.85 (-1.12 to -0.59)
Barbados	8 (6 to 11)	3.59 (2.67 to 4.64)	2.55 (1.91 to 3.33)	7 (5 to 9)	2.95 (2.10 to 3.97)	1.30 (0.86 to 1.80)	-16.84 (-40.25 to 14.69)	-2.94 (-3.26 to -2.63)
Belarus	1267 (982 to 1520)	9.86 (7.94 to 11.86)	9.75 (7.57 to 11.77)	1215 (920 to 1539)	9.85 (8.17 to 11.79)	7.38 (5.59 to 9.36)	-4.06 (-23.96 to 22.49)	-1.57 (-2.04 to -1.09)
Belgium	924 (700 to 1172)	10.13 (7.86 to 12.84)	5.75 (4.37 to 7.27)	288 (212 to 385)	6.06 (4.47 to 8.16)	1.03 (0.79 to 1.35)	-68.79 (-74.40 to -61.04)	-5.48 (-5.57 to -5.39)
Belize	1 (1 to 2)	5.38 (4.16 to 6.94)	1.58 (1.20 to 2.04)	3 (2 to 4)	5.03 (3.84 to 6.51)	1.20 (0.86 to 1.59)	121.19 (70.48 to 184.51)	-1.13 (-1.6 to -0.65)
Benin	54 (39 to 74)	3.77 (2.80 to 4.86)	2.86 (2.01 to 3.91)	69 (47 to 98)	2.44 (1.79 to 3.26)	1.47 (1.01 to 2.09)	28.01 (-13.05 to 85.48)	-2.31 (-2.45 to -2.17)
Bermuda	1 (1 to 2)	5.42 (4.06 to 6.96)	2.49 (1.77 to 3.35)	2 (1 to 2)	4.74 (3.37 to 6.54)	1.04 (0.73 to 1.45)	3.33 (-23.48 to 44.39)	-2.73 (-3.01 to -2.44)
Bhutan	4 (2 to 6)	5.52 (3.88 to 7.76)	2.22 (1.17 to 3.58)	10 (6 to 14)	4.83 (3.39 to 6.75)	1.78 (1.16 to 2.67)	148.75 (60.63 to 314.10)	-0.72 (-0.8 to -0.63)
Bolivia (Plurinational State of)	63 (40 to 86)	5.24 (4.01 to 6.83)	2.23 (1.43 to 3.08)	84 (54 to 126)	3.94 (3.03 to 5.15)	1.04 (0.67 to 1.57)	33.43 (-9.96 to 92.94)	-2.18 (-2.5 to -1.86)

Location	1990			2021			1990-2021	
	Deaths Cases (95%UI)	PAF, % (95%UI)	ASMR (95%UI)	Deaths Cases (95%UI)	PAF, % (95%UI)	ASMR (95%UI)	Cases change, % (95%UI)	EAPC (95%CI)
Bosnia and Herzegovina	471 (366 to 582)	12.34 (9.93 to 14.90)	13.29 (10.25 to 16.62)	621 (438 to 821)	10.59 (8.17 to 13.10)	9.60 (6.77 to 12.66)	31.77 (-5.36 to 79.19)	-1.21 (-1.51 to -0.91)
Botswana	33 (23 to 44)	11.43 (8.95 to 14.20)	7.39 (5.08 to 10.07)	45 (32 to 64)	8.29 (6.36 to 10.35)	3.79 (2.69 to 5.36)	37.03 (-4.75 to 101.41)	-2.28 (-2.46 to -2.11)
Brazil	7935 (6543 to 9549)	14.64 (12.12 to 17.63)	10.15 (8.12 to 12.55)	5232 (4056 to 6575)	7.53 (5.89 to 9.44)	2.14 (1.65 to 2.71)	-34.07 (-42.90 to -25.06)	-5.08 (-5.32 to -4.84)
Brunei Darussalam	7 (5 to 9)	13.36 (10.57 to 16.13)	8.52 (6.27 to 10.92)	5 (4 to 7)	7.95 (6.12 to 10.33)	2.16 (1.52 to 2.93)	-23.39 (-44.13 to 3.87)	-4.13 (-4.45 to -3.8)
Bulgaria	1597 (1310 to 1922)	10.33 (8.51 to 12.43)	13.78 (11.10 to 16.77)	1307 (1003 to 1638)	5.99 (4.84 to 7.27)	9.20 (7.09 to 11.51)	-18.17 (-32.26 to -0.73)	-1.2 (-1.39 to -1)
Burkina Faso	47 (30 to 72)	3.32 (2.48 to 4.44)	1.18 (0.75 to 1.77)	86 (56 to 127)	2.76 (1.99 to 3.71)	1.00 (0.64 to 1.44)	85.32 (30.11 to 172.86)	-0.36 (-0.53 to -0.18)
Burundi	64 (41 to 99)	4.33 (3.14 to 5.75)	3.16 (2.04 to 4.92)	42 (26 to 64)	2.64 (1.92 to 3.67)	1.04 (0.65 to 1.62)	-35.14 (-57.88 to 1.12)	-3.9 (-4.51 to -3.28)
Cabo Verde	4 (3 to 6)	3.37 (2.59 to 4.38)	1.81 (1.27 to 2.53)	6 (5 to 9)	2.37 (1.84 to 3.06)	1.44 (1.04 to 1.94)	54.76 (10.28 to 125.51)	-1.45 (-1.99 to -0.91)
Cambodia	296 (218 to 399)	13.20 (10.39 to 16.14)	8.77 (6.24 to 11.68)	745 (516 to 1005)	12.40 (9.53 to 15.95)	8.11 (5.55 to 11.06)	151.34 (79.11 to 238.74)	-0.47 (-0.63 to -0.3)
Cameroon	86 (58 to 127)	4.41 (3.30 to 5.90)	2.13 (1.45 to 3.13)	217 (133 to 333)	3.62 (2.70 to 4.66)	1.86 (1.17 to 2.89)	151.62 (61.17 to 278.33)	-0.36 (-0.94 to 0.21)
Canada	1160 (879 to 1481)	10.98 (8.51 to 13.96)	3.60 (2.71 to 4.60)	638 (442 to 866)	5.93 (4.14 to 7.96)	0.77 (0.55 to 1.03)	-44.98 (-56.14 to -31.62)	-5.48 (-5.76 to -5.2)
Central African Republic	24 (15 to 38)	4.78 (3.37 to 6.52)	2.56 (1.63 to 4.02)	29 (18 to 48)	3.66 (2.56 to 5.06)	1.63 (1.04 to 2.56)	21.04 (-19.75 to 75.70)	-1.62 (-1.81 to -1.42)

Location	1990			2021			1990-2021	
	**Deaths Cases (95%UI)**	**PAF, % (95%UI)**	**ASMR (95%UI)**	**Deaths Cases (95%UI)**	**PAF, % (95%UI)**	**ASMR (95%UI)**	**Cases change, % (95%UI)**	**EAPC (95%CI)**
Chad	73 (45 to 111)	4.62 (3.22 to 6.11)	2.81 (1.71 to 4.21)	135 (85 to 213)	4.20 (2.95 to 5.70)	2.74 (1.68 to 4.24)	84.71 (20.99 to 199.27)	-0.38 (-0.62 to -0.14)
Chile	257 (208 to 326)	5.29 (4.29 to 6.62)	2.65 (2.11 to 3.38)	177 (135 to 233)	2.99 (2.28 to 3.88)	0.68 (0.52 to 0.89)	-31.06 (-41.41 to -18.84)	-4.11 (-4.24 to -3.97)
China	74875 (59572 to 96058)	17.48 (14.47 to 20.97)	10.83 (8.38 to 14.02)	165387 (122395 to 218443)	14.04 (11.17 to 17.33)	8.35 (6.13 to 10.97)	120.88 (59.72 to 203.10)	-0.67 (-0.85 to -0.49)
Colombia	323 (263 to 397)	6.00 (4.87 to 7.32)	2.00 (1.59 to 2.47)	264 (193 to 362)	3.23 (2.50 to 4.11)	0.48 (0.35 to 0.65)	-18.39 (-38.22 to 3.33)	-5.36 (-5.73 to -4.99)
Comoros	5 (3 to 8)	6.31 (4.48 to 8.78)	3.55 (2.25 to 5.33)	8 (5 to 11)	4.40 (2.99 to 6.10)	1.95 (1.16 to 2.99)	40.45 (-4.63 to 117.83)	-2.37 (-2.62 to -2.12)
Congo	24 (15 to 34)	4.37 (3.03 to 5.87)	2.64 (1.73 to 3.70)	50 (33 to 71)	4.54 (3.30 to 6.18)	2.36 (1.60 to 3.41)	105.72 (42.29 to 189.46)	-0.43 (-0.7 to -0.15)
Cook Islands	0 (0 to 0)	7.85 (6.01 to 9.98)	2.79 (1.97 to 3.71)	0 (0 to 0)	5.59 (4.17 to 7.40)	1.28 (0.88 to 1.78)	5.72 (-23.81 to 45.31)	-2.73 (-2.91 to -2.54)
Costa Rica	30 (23 to 38)	7.12 (5.56 to 9.02)	1.83 (1.40 to 2.33)	41 (30 to 53)	4.52 (3.40 to 5.66)	0.74 (0.55 to 0.97)	36.84 (8.16 to 65.31)	-3.39 (-3.68 to -3.1)
Croatia	893 (694 to 1123)	12.83 (10.10 to 16.09)	16.85 (12.94 to 21.43)	459 (337 to 613)	9.16 (6.90 to 12.00)	4.53 (3.36 to 6.02)	-48.64 (-59.65 to -34.35)	-4.02 (-4.17 to -3.87)
Cuba	423 (334 to 540)	10.39 (8.37 to 13.25)	4.26 (3.31 to 5.47)	543 (410 to 696)	7.38 (5.80 to 9.29)	2.69 (2.04 to 3.43)	28.26 (2.54 to 62.33)	-1.57 (-1.71 to -1.43)
Cyprus	41 (29 to 54)	7.39 (5.37 to 9.88)	7.58 (5.13 to 10.58)	34 (24 to 47)	6.70 (4.86 to 8.91)	1.74 (1.20 to 2.43)	-16.02 (-38.53 to 13.97)	-5.14 (-5.31 to -4.96)
Czechia	2188 (1703 to 2754)	10.40 (8.24 to 12.96)	15.58 (12.02 to 19.69)	528 (397 to 672)	7.23 (5.53 to 9.00)	2.30 (1.76 to 2.91)	-75.88 (-80.94 to -70.58)	-6.53 (-6.86 to -6.19)

Location	1990			2021			1990-2021	
	Deaths Cases (95%UI)	PAF, % (95%UI)	ASMR (95%UI)	Deaths Cases (95%UI)	PAF, % (95%UI)	ASMR (95%UI)	Cases change, % (95%UI)	EAPC (95%CI)
Côte d'Ivoire	136 (96 to 189)	6.58 (5.06 to 8.30)	4.00 (2.85 to 5.53)	292 (189 to 436)	5.09 (3.86 to 6.54)	2.89 (1.92 to 4.16)	115.04 (44.71 to 215.58)	-1.77 (-2.22 to -1.31)
Democratic People's Republic of Korea	939 (659 to 1304)	11.73 (9.21 to 14.60)	6.35 (4.53 to 8.72)	1876 (1377 to 2550)	9.98 (7.85 to 12.31)	5.72 (4.21 to 7.79)	99.85 (47.79 to 181.49)	-0.52 (-0.69 to -0.35)
Democratic Republic of the Congo	168 (114 to 248)	2.98 (2.17 to 4.03)	1.31 (0.88 to 1.96)	256 (156 to 433)	2.13 (1.46 to 3.08)	0.82 (0.49 to 1.39)	52.56 (-0.87 to 133.18)	-1.57 (-1.88 to -1.25)
Denmark	770 (598 to 948)	17.52 (13.74 to 21.43)	8.67 (6.78 to 10.62)	259 (186 to 339)	9.63 (7.25 to 12.52)	1.87 (1.35 to 2.43)	-66.41 (-71.31 to -60.28)	-5.46 (-5.79 to -5.13)
Djibouti	4 (2 to 6)	9.54 (6.97 to 12.60)	4.24 (2.54 to 7.00)	17 (11 to 26)	8.90 (6.68 to 11.68)	3.75 (2.40 to 5.56)	338.87 (162.45 to 630.03)	-0.44 (-0.49 to -0.39)
Dominica	1 (1 to 2)	3.32 (2.52 to 4.33)	2.07 (1.55 to 2.76)	1 (1 to 2)	3.00 (2.25 to 4.00)	1.47 (1.04 to 2.06)	-4.26 (-25.65 to 24.52)	-1.16 (-1.48 to -0.83)
Dominican Republic	134 (98 to 174)	11.70 (9.10 to 14.76)	4.47 (3.21 to 6.06)	307 (204 to 440)	9.41 (6.98 to 12.25)	3.20 (2.11 to 4.58)	129.22 (55.91 to 224.87)	-0.71 (-1.11 to -0.31)
Ecuador	117 (91 to 145)	7.29 (5.95 to 8.94)	2.40 (1.84 to 3.01)	100 (70 to 136)	3.59 (2.75 to 4.61)	0.64 (0.45 to 0.88)	-14.09 (-37.06 to 13.86)	-4.08 (-4.4 to -3.75)
Egypt	2955 (1975 to 4447)	9.74 (7.02 to 13.31)	14.52 (9.64 to 21.55)	6304 (4515 to 8741)	11.52 (9.31 to 14.06)	12.89 (9.14 to 17.75)	113.33 (43.90 to 205.80)	-0.07 (-0.3 to 0.17)
El Salvador	23 (17 to 29)	3.29 (2.52 to 4.25)	0.79 (0.58 to 1.03)	31 (21 to 43)	2.83 (2.13 to 3.68)	0.49 (0.34 to 0.68)	35.66 (-0.17 to 83.14)	-1.75 (-2.09 to -1.41)
Equatorial Guinea	4 (2 to 6)	4.48 (3.13 to 6.55)	2.40 (1.50 to 3.77)	6 (3 to 10)	3.27 (2.23 to 4.61)	1.43 (0.83 to 2.26)	49.70 (-18.07 to 174.87)	-2.01 (-2.4 to -1.61)
Eritrea	11 (6 to 21)	3.24 (2.04 to 5.07)	1.11 (0.62 to 2.02)	18 (12 to 29)	2.23 (1.47 to 3.20)	0.78 (0.49 to 1.21)	68.44 (2.17 to 189.02)	-1.47 (-1.68 to -1.27)

Location	1990			2021			1990-2021	
	**Deaths Cases (95%UI)**	**PAF, % (95%UI)**	**ASMR (95%UI)**	**Deaths Cases (95%UI)**	**PAF, % (95%UI)**	**ASMR (95%UI)**	**Cases change, % (95%UI)**	**EAPC (95%CI)**
Estonia	213 (173 to 262)	7.90 (6.47 to 9.62)	10.31 (8.33 to 12.63)	55 (43 to 70)	6.22 (4.99 to 7.85)	2.01 (1.59 to 2.53)	-74.22 (-78.68 to -69.10)	-6.7 (-7.39 to -6.01)
Eswatini	7 (5 to 10)	5.59 (4.22 to 7.22)	3.61 (2.34 to 5.12)	10 (6 to 15)	3.97 (2.91 to 5.36)	2.50 (1.49 to 3.95)	37.18 (-17.75 to 121.58)	-0.85 (-1.23 to -0.48)
Ethiopia	101 (54 to 189)	2.45 (1.51 to 3.67)	0.57 (0.30 to 1.04)	139 (90 to 210)	1.63 (1.16 to 2.23)	0.38 (0.24 to 0.57)	37.38 (-24.14 to 127.06)	-1.06 (-1.34 to -0.78)
Fiji	10 (7 to 13)	9.70 (7.88 to 11.69)	3.10 (2.31 to 4.07)	13 (10 to 18)	6.68 (5.38 to 8.25)	1.93 (1.41 to 2.62)	39.01 (-0.03 to 97.70)	-1.97 (-2.23 to -1.71)
Finland	259 (206 to 323)	6.08 (4.85 to 7.58)	3.58 (2.86 to 4.43)	120 (85 to 162)	3.71 (2.68 to 4.90)	0.82 (0.60 to 1.08)	-53.89 (-63.35 to -43.54)	-4.64 (-4.71 to -4.57)
France	3134 (2352 to 4046)	7.69 (5.83 to 9.88)	3.47 (2.65 to 4.47)	1250 (895 to 1698)	4.39 (3.15 to 5.85)	0.72 (0.55 to 0.95)	-60.11 (-67.38 to -51.10)	-5.06 (-5.19 to -4.93)
Gabon	8 (5 to 11)	2.71 (1.99 to 3.61)	1.44 (0.99 to 2.05)	13 (8 to 18)	3.18 (2.24 to 4.35)	1.37 (0.90 to 1.96)	64.92 (7.80 to 140.91)	-0.22 (-0.3 to -0.13)
Gambia	17 (12 to 24)	8.50 (6.63 to 10.68)	5.20 (3.53 to 7.34)	30 (20 to 43)	4.55 (3.38 to 5.94)	3.31 (2.22 to 4.62)	78.97 (17.09 to 159.67)	-1.73 (-1.9 to -1.56)
Georgia	326 (217 to 439)	7.62 (5.59 to 9.74)	5.40 (3.60 to 7.29)	585 (460 to 730)	9.19 (7.32 to 11.26)	9.34 (7.47 to 11.55)	79.58 (39.16 to 156.53)	2.36 (1.9 to 2.82)
Germany	8460 (6436 to 10743)	9.20 (7.08 to 11.95)	6.25 (4.78 to 7.86)	2676 (1972 to 3566)	6.10 (4.61 to 8.15)	1.22 (0.94 to 1.58)	-68.37 (-73.90 to -61.61)	-5.38 (-5.75 to -5.01)
Ghana	171 (123 to 231)	4.11 (3.16 to 5.32)	3.47 (2.44 to 4.61)	518 (367 to 693)	4.43 (3.35 to 5.74)	3.78 (2.62 to 5.12)	203.37 (104.61 to 345.62)	1.07 (0.78 to 1.36)
Greece	1573 (1201 to 1959)	10.88 (8.32 to 13.72)	10.69 (8.09 to 13.44)	754 (534 to 1028)	6.92 (4.90 to 9.16)	2.31 (1.70 to 3.03)	-52.08 (-61.40 to -40.76)	-5.59 (-5.89 to -5.29)

Location	1990			2021			1990-2021	
	Deaths Cases (95%UI)	PAF, % (95%UI)	ASMR (95%UI)	Deaths Cases (95%UI)	PAF, % (95%UI)	ASMR (95%UI)	Cases change, % (95%UI)	EAPC (95%CI)
Greenland	3 (2 to 3)	14.87 (11.87 to 18.47)	10.84 (8.02 to 14.66)	2 (1 to 2)	11.24 (8.76 to 14.52)	2.99 (2.14 to 4.21)	-38.19 (-55.74 to -16.00)	-4.41 (-4.59 to -4.23)
Grenada	3 (2 to 4)	3.82 (2.96 to 4.95)	3.87 (2.94 to 5.03)	1 (1 to 2)	3.26 (2.52 to 4.19)	1.36 (0.98 to 1.81)	-50.00 (-59.97 to -35.15)	-3.53 (-3.8 to -3.26)
Guam	1 (1 to 2)	7.22 (5.87 to 9.22)	1.84 (1.41 to 2.44)	2 (1 to 2)	7.32 (5.75 to 9.28)	0.89 (0.68 to 1.16)	44.68 (8.23 to 99.00)	-2.25 (-2.58 to -1.91)
Guatemala	34 (25 to 44)	5.10 (3.94 to 6.49)	1.32 (0.96 to 1.75)	61 (43 to 84)	3.69 (2.70 to 4.98)	0.63 (0.43 to 0.86)	79.36 (28.18 to 149.14)	-3.08 (-3.42 to -2.73)
Guinea	88 (59 to 122)	4.65 (3.47 to 6.13)	2.90 (1.91 to 3.99)	158 (104 to 222)	4.55 (3.35 to 5.95)	3.07 (2.01 to 4.28)	80.38 (19.45 to 184.95)	0.37 (0.2 to 0.55)
Guinea-Bissau	9 (6 to 13)	2.99 (2.26 to 3.90)	2.42 (1.61 to 3.48)	17 (12 to 24)	3.63 (2.69 to 4.73)	2.45 (1.70 to 3.36)	89.09 (28.16 to 181.12)	0.51 (0.28 to 0.75)
Guyana	21 (17 to 27)	6.00 (4.84 to 7.45)	5.88 (4.64 to 7.56)	16 (11 to 22)	4.53 (3.47 to 5.81)	2.65 (1.83 to 3.70)	-25.85 (-46.98 to 1.88)	-1.99 (-2.19 to -1.79)
Haiti	82 (57 to 113)	3.45 (2.62 to 4.44)	2.88 (1.99 to 3.97)	102 (63 to 155)	2.56 (1.92 to 3.38)	1.72 (1.07 to 2.65)	24.70 (-12.56 to 81.34)	-1.52 (-1.72 to -1.31)
Honduras	50 (34 to 68)	7.59 (5.87 to 9.61)	2.73 (1.85 to 3.76)	160 (106 to 218)	6.15 (4.59 to 7.96)	2.89 (1.95 to 3.95)	220.19 (128.86 to 355.75)	0.54 (0.27 to 0.8)
Hungary	1930 (1610 to 2329)	10.72 (8.93 to 12.90)	13.14 (10.99 to 15.88)	605 (477 to 763)	6.49 (5.24 to 7.92)	3.09 (2.47 to 3.83)	-68.64 (-73.89 to -62.47)	-5.12 (-5.35 to -4.89)
Iceland	14 (10 to 18)	10.98 (8.29 to 14.37)	4.44 (3.32 to 5.82)	5 (3 to 7)	5.21 (3.52 to 7.33)	0.75 (0.51 to 1.07)	-62.06 (-71.97 to -48.77)	-5.93 (-6.15 to -5.71)
India	12737 (9049 to 18428)	10.64 (8.35 to 13.40)	3.51 (2.49 to 4.96)	23980 (17734 to 34954)	7.64 (5.89 to 9.81)	2.31 (1.70 to 3.25)	88.27 (44.69 to 137.31)	-1.46 (-1.59 to -1.33)

Location	1990			2021			1990-2021	
	Deaths Cases (95%UI)	PAF, % (95%UI)	ASMR (95%UI)	Deaths Cases (95%UI)	PAF, % (95%UI)	ASMR (95%UI)	Cases change, % (95%UI)	EAPC (95%CI)
Indonesia	4733 (3540 to 6076)	10.09 (7.68 to 12.83)	6.15 (4.59 to 7.99)	16835 (10621 to 23706)	11.31 (8.37 to 14.28)	9.04 (5.93 to 12.40)	255.71 (148.39 to 391.09)	1.36 (1.08 to 1.64)
Iran (Islamic Republic of)	1397 (1123 to 1726)	8.26 (6.75 to 9.96)	5.89 (4.60 to 7.40)	2240 (1787 to 2803)	6.59 (5.29 to 8.16)	3.06 (2.37 to 3.88)	60.32 (34.12 to 90.81)	-2 (-2.12 to -1.87)
Iraq	1395 (1038 to 1744)	15.52 (12.58 to 18.62)	19.05 (14.07 to 23.99)	2601 (1860 to 3450)	12.73 (10.27 to 15.41)	13.68 (9.84 to 18.36)	86.38 (30.68 to 151.96)	-1.73 (-1.95 to -1.51)
Ireland	368 (286 to 459)	15.46 (12.11 to 18.92)	9.18 (6.99 to 11.51)	83 (55 to 114)	6.53 (4.58 to 8.81)	0.95 (0.64 to 1.31)	-77.54 (-82.66 to -70.90)	-7.52 (-7.8 to -7.24)
Israel	145 (111 to 187)	9.32 (7.14 to 11.73)	3.09 (2.34 to 4.02)	78 (55 to 103)	4.89 (3.61 to 6.42)	0.56 (0.41 to 0.75)	-46.38 (-58.56 to -31.27)	-6.09 (-6.31 to -5.86)
Italy	5226 (4015 to 6537)	9.13 (7.02 to 11.41)	5.79 (4.43 to 7.26)	1731 (1207 to 2297)	4.16 (2.95 to 5.52)	0.92 (0.67 to 1.20)	-66.88 (-73.77 to -59.50)	-6.29 (-6.62 to -5.97)
Jamaica	69 (53 to 88)	5.47 (4.21 to 6.87)	3.74 (2.90 to 4.74)	66 (47 to 95)	4.09 (3.04 to 5.44)	2.11 (1.49 to 3.02)	-4.22 (-29.17 to 29.00)	-1.77 (-2.23 to -1.3)
Japan	9335 (7543 to 11496)	11.24 (9.04 to 13.77)	5.85 (4.68 to 7.26)	4661 (3163 to 6282)	5.27 (3.79 to 7.00)	0.99 (0.74 to 1.27)	-50.07 (-59.93 to -39.26)	-6.2 (-6.42 to -5.98)
Jordan	143 (105 to 185)	13.91 (11.37 to 16.76)	13.58 (9.78 to 17.74)	323 (239 to 428)	12.28 (10.01 to 15.01)	5.56 (4.04 to 7.53)	126.54 (68.48 to 208.53)	-3.35 (-3.59 to -3.11)
Kazakhstan	988 (778 to 1196)	7.84 (6.35 to 9.26)	7.86 (6.16 to 9.64)	916 (733 to 1121)	6.95 (5.68 to 8.32)	5.27 (4.17 to 6.47)	-7.35 (-24.96 to 16.78)	-1.84 (-2.56 to -1.12)
Kenya	110 (72 to 160)	5.20 (3.74 to 6.85)	1.74 (1.10 to 2.54)	201 (139 to 286)	3.49 (2.44 to 4.66)	1.15 (0.78 to 1.61)	83.00 (28.83 to 161.01)	-1.75 (-1.89 to -1.62)
Kiribati	2 (2 to 3)	17.21 (14.59 to 20.06)	6.71 (4.90 to 8.72)	5 (3 to 6)	19.24 (16.14 to 22.83)	7.48 (5.40 to 10.08)	111.38 (57.73 to 210.00)	0.18 (-0.07 to 0.42)

Location	1990			2021			1990-2021	
	Deaths Cases (95%UI)	PAF, % (95%UI)	ASMR (95%UI)	Deaths Cases (95%UI)	PAF, % (95%UI)	ASMR (95%UI)	Cases change, % (95%UI)	EAPC (95%CI)
Kuwait	17 (14 to 21)	11.63 (9.66 to 13.80)	3.35 (2.58 to 4.16)	64 (47 to 85)	11.87 (9.44 to 14.67)	2.59 (1.80 to 3.53)	275.25 (185.79 to 379.44)	-0.62 (-1.73 to 0.5)
Kyrgyzstan	249 (194 to 307)	8.54 (6.87 to 10.39)	8.83 (6.87 to 10.95)	389 (305 to 482)	16.74 (14.15 to 19.56)	8.43 (6.66 to 10.54)	56.01 (24.30 to 104.67)	0.11 (-0.27 to 0.48)
Lao People's Democratic Republic	200 (144 to 266)	12.97 (10.36 to 16.00)	11.49 (8.38 to 15.19)	314 (214 to 428)	12.55 (9.84 to 15.45)	8.56 (5.79 to 11.76)	56.79 (5.95 to 127.25)	-0.97 (-1.04 to -0.9)
Latvia	390 (314 to 476)	7.53 (6.14 to 9.03)	10.83 (8.67 to 13.23)	208 (158 to 266)	4.84 (3.81 to 5.97)	5.11 (3.97 to 6.44)	-46.61 (-56.98 to -35.44)	-2.83 (-3.12 to -2.55)
Lebanon	175 (127 to 239)	13.96 (11.34 to 16.87)	9.18 (6.56 to 12.62)	239 (169 to 321)	12.51 (9.59 to 16.20)	3.69 (2.65 to 4.89)	36.52 (-6.94 to 93.00)	-3.13 (-3.33 to -2.94)
Lesotho	28 (19 to 41)	7.61 (5.53 to 10.12)	4.04 (2.62 to 6.20)	58 (36 to 89)	8.89 (6.32 to 12.09)	6.83 (4.16 to 10.82)	106.08 (23.38 to 242.44)	2.61 (2.23 to 2.98)
Liberia	24 (17 to 32)	3.40 (2.65 to 4.28)	2.23 (1.60 to 3.01)	33 (22 to 48)	3.10 (2.31 to 4.06)	1.63 (1.08 to 2.37)	39.29 (-3.65 to 109.15)	-1.33 (-1.5 to -1.15)
Libya	64 (43 to 92)	7.59 (5.70 to 9.69)	3.78 (2.52 to 5.47)	192 (123 to 283)	8.16 (6.12 to 10.73)	4.01 (2.59 to 6.00)	199.94 (104.22 to 352.94)	0.75 (0.51 to 0.98)
Lithuania	294 (238 to 357)	8.62 (7.05 to 10.46)	6.51 (5.29 to 7.91)	221 (172 to 281)	5.84 (4.59 to 7.23)	3.68 (2.92 to 4.62)	-24.95 (-39.52 to -10.71)	-2.03 (-2.37 to -1.69)
Luxembourg	40 (30 to 53)	8.08 (5.98 to 10.69)	7.14 (5.31 to 9.61)	11 (8 to 16)	5.56 (3.94 to 7.76)	0.96 (0.68 to 1.32)	-71.62 (-78.21 to -62.29)	-6.43 (-6.57 to -6.29)
Madagascar	154 (108 to 220)	5.62 (4.22 to 7.39)	3.97 (2.69 to 5.72)	127 (81 to 190)	2.83 (2.11 to 3.81)	1.60 (1.01 to 2.37)	-17.79 (-44.54 to 20.55)	-3.17 (-3.55 to -2.79)
Malawi	103 (69 to 142)	7.06 (4.89 to 9.49)	3.69 (2.41 to 5.12)	227 (146 to 339)	7.06 (4.87 to 9.74)	3.84 (2.47 to 5.61)	120.74 (55.60 to 213.51)	-0.1 (-0.34 to 0.14)

Location	1990			2021			1990-2021	
	Deaths Cases (95%UI)	PAF, % (95%UI)	ASMR (95%UI)	Deaths Cases (95%UI)	PAF, % (95%UI)	ASMR (95%UI)	Cases change, % (95%UI)	EAPC (95%CI)
Malaysia	445 (343 to 577)	10.96 (8.73 to 13.58)	5.40 (4.12 to 7.05)	727 (565 to 906)	7.68 (6.14 to 9.50)	2.90 (2.22 to 3.70)	63.38 (25.08 to 110.50)	-2.02 (-2.19 to -1.86)
Maldives	7 (5 to 9)	17.08 (13.73 to 20.62)	12.64 (8.99 to 16.69)	12 (8 to 15)	11.64 (9.01 to 14.67)	4.45 (3.08 to 5.88)	61.27 (18.08 to 117.99)	-3.74 (-3.87 to -3.61)
Mali	45 (28 to 69)	3.02 (2.07 to 4.12)	1.49 (0.91 to 2.19)	157 (104 to 227)	4.86 (3.46 to 6.42)	2.32 (1.57 to 3.34)	246.49 (136.86 to 425.26)	2.04 (1.79 to 2.3)
Malta	20 (16 to 25)	8.66 (6.71 to 10.81)	4.88 (3.73 to 6.19)	7 (5 to 10)	4.04 (2.87 to 5.51)	0.66 (0.46 to 0.90)	-64.39 (-72.30 to -54.83)	-6.58 (-6.79 to -6.36)
Marshall Islands	0 (0 to 1)	6.57 (4.72 to 8.96)	3.05 (2.00 to 4.66)	1 (0 to 1)	7.79 (5.84 to 10.12)	2.66 (1.67 to 4.14)	73.65 (20.26 to 146.24)	-0.37 (-0.44 to -0.31)
Mauritania	28 (19 to 41)	4.01 (2.98 to 5.40)	2.89 (1.98 to 4.22)	33 (21 to 52)	2.76 (2.00 to 3.73)	1.63 (1.04 to 2.54)	19.18 (-16.69 to 71.13)	-2.12 (-2.39 to -1.86)
Mauritius	73 (61 to 86)	12.65 (10.74 to 14.83)	10.53 (8.65 to 12.62)	57 (46 to 70)	9.71 (7.86 to 11.78)	3.23 (2.57 to 3.96)	-21.71 (-33.60 to -7.98)	-4.94 (-5.55 to -4.33)
Mexico	1123 (896 to 1363)	8.63 (6.85 to 10.47)	3.31 (2.52 to 4.13)	815 (614 to 1042)	4.17 (3.22 to 5.17)	0.69 (0.51 to 0.90)	-27.44 (-42.36 to -10.67)	-5.32 (-5.48 to -5.17)
Micronesia (Federated States of)	2 (2 to 3)	9.93 (7.63 to 12.69)	5.09 (3.40 to 7.43)	3 (2 to 4)	11.28 (8.82 to 13.81)	3.95 (2.64 to 5.89)	17.85 (-18.65 to 69.23)	-0.96 (-1.05 to -0.88)
Monaco	5 (3 to 7)	7.56 (5.49 to 10.28)	6.34 (4.34 to 8.99)	2 (1 to 3)	5.46 (3.69 to 7.65)	1.95 (1.28 to 2.85)	-55.67 (-69.92 to -34.35)	-3.99 (-4.19 to -3.79)
Mongolia	19 (13 to 26)	9.84 (7.84 to 12.08)	1.93 (1.34 to 2.65)	53 (34 to 73)	14.18 (11.06 to 17.50)	2.47 (1.58 to 3.44)	175.09 (67.14 to 316.78)	1.13 (0.7 to 1.56)
Montenegro	23 (17 to 32)	10.87 (8.55 to 13.35)	3.98 (2.82 to 5.36)	44 (30 to 61)	8.40 (6.27 to 10.83)	4.61 (3.09 to 6.48)	87.56 (25.06 to 171.58)	0.61 (0.39 to 0.84)

Location	1990			2021			1990-2021	
	**Deaths Cases (95%UI)**	**PAF, % (95%UI)**	**ASMR (95%UI)**	**Deaths Cases (95%UI)**	**PAF, % (95%UI)**	**ASMR (95%UI)**	**Cases change, % (95%UI)**	**EAPC (95%CI)**
Morocco	658 (443 to 929)	5.72 (4.22 to 7.56)	4.95 (3.30 to 7.04)	1008 (672 to 1409)	3.91 (2.96 to 5.16)	3.06 (2.06 to 4.27)	53.15 (8.17 to 114.15)	-1.59 (-1.75 to -1.43)
Mozambique	168 (121 to 225)	5.58 (4.22 to 7.02)	3.46 (2.44 to 4.71)	375 (235 to 537)	5.57 (4.19 to 7.45)	3.88 (2.53 to 5.49)	123.68 (44.37 to 218.01)	0.91 (0.73 to 1.1)
Myanmar	3043 (2167 to 4003)	18.40 (15.09 to 22.04)	16.41 (11.71 to 21.48)	2563 (1809 to 3469)	9.37 (7.27 to 11.96)	6.29 (4.41 to 8.51)	-15.76 (-40.42 to 19.79)	-3.34 (-3.45 to -3.23)
Namibia	38 (29 to 51)	10.73 (8.48 to 13.37)	8.74 (6.23 to 12.08)	53 (37 to 72)	7.27 (5.51 to 9.18)	5.42 (3.68 to 7.61)	39.52 (-1.52 to 93.61)	-1.93 (-2.25 to -1.6)
Nauru	0 (0 to 0)	13.17 (10.59 to 16.05)	7.96 (5.23 to 10.86)	0 (0 to 0)	10.57 (8.45 to 13.07)	5.67 (3.98 to 7.80)	-2.18 (-31.73 to 42.12)	-1.33 (-1.65 to -1.01)
Nepal	602 (398 to 853)	17.28 (14.01 to 20.63)	8.57 (5.72 to 12.37)	877 (587 to 1282)	11.82 (9.27 to 14.78)	4.61 (3.04 to 6.72)	45.70 (3.70 to 110.46)	-2.23 (-2.41 to -2.04)
Netherlands	1070 (814 to 1366)	11.02 (8.35 to 13.93)	5.16 (3.93 to 6.59)	610 (433 to 817)	7.12 (5.18 to 9.55)	1.52 (1.09 to 2.01)	-42.98 (-53.76 to -29.32)	-4.2 (-4.44 to -3.95)
New Zealand	146 (113 to 186)	8.58 (6.75 to 10.85)	3.67 (2.81 to 4.68)	97 (65 to 133)	5.29 (3.70 to 7.12)	1.03 (0.71 to 1.41)	-33.71 (-47.65 to -18.12)	-4.2 (-4.47 to -3.94)
Nicaragua	22 (17 to 29)	6.12 (4.79 to 7.80)	1.67 (1.24 to 2.22)	35 (24 to 50)	5.17 (3.88 to 6.74)	0.79 (0.54 to 1.13)	57.97 (12.92 to 114.08)	-2.13 (-2.32 to -1.94)
Niger	23 (14 to 37)	2.32 (1.56 to 3.11)	1.19 (0.72 to 1.81)	66 (40 to 104)	2.19 (1.49 to 3.15)	1.20 (0.70 to 1.87)	180.66 (89.35 to 313.32)	0.04 (-0.07 to 0.14)
Nigeria	660 (450 to 971)	2.55 (1.95 to 3.36)	1.75 (1.19 to 2.52)	809 (529 to 1168)	2.12 (1.53 to 2.76)	1.00 (0.66 to 1.43)	22.58 (-27.11 to 110.46)	-1.92 (-2.17 to -1.67)
Niue	0 (0 to 0)	4.47 (3.27 to 5.84)	2.92 (2.11 to 4.00)	0 (0 to 0)	5.29 (3.91 to 7.02)	2.32 (1.60 to 3.20)	-24.18 (-44.85 to 6.64)	-1.04 (-1.16 to -0.92)

Location	1990			2021			1990-2021	
	**Deaths Cases (95%UI)**	**PAF, % (95%UI)**	**ASMR (95%UI)**	**Deaths Cases (95%UI)**	**PAF, % (95%UI)**	**ASMR (95%UI)**	**Cases change, % (95%UI)**	**EAPC (95%CI)**
North Macedonia	371 (285 to 474)	12.53 (10.09 to 15.70)	22.29 (16.90 to 28.95)	470 (341 to 638)	9.61 (7.49 to 11.95)	17.35 (11.98 to 24.40)	26.72 (-8.64 to 73.37)	-1.09 (-1.59 to -0.59)
Northern Mariana Islands	0 (0 to 1)	10.91 (8.62 to 13.35)	3.15 (2.28 to 4.24)	1 (1 to 1)	8.20 (6.43 to 10.28)	1.84 (1.36 to 2.49)	94.00 (32.78 to 167.11)	-2.19 (-2.43 to -1.94)
Norway	413 (306 to 545)	9.36 (7.03 to 12.34)	5.38 (4.05 to 7.04)	79 (53 to 113)	4.26 (2.92 to 5.91)	0.66 (0.46 to 0.93)	-80.97 (-86.15 to -73.98)	-7.29 (-7.61 to -6.97)
Oman	30 (21 to 41)	7.46 (6.09 to 9.14)	4.89 (3.36 to 6.65)	40 (28 to 53)	5.90 (4.67 to 7.22)	2.42 (1.71 to 3.37)	31.64 (-9.47 to 105.93)	-1.6 (-1.93 to -1.28)
Pakistan	2423 (1451 to 3711)	11.50 (8.51 to 14.63)	4.99 (3.01 to 7.61)	3312 (2304 to 4672)	8.05 (6.04 to 10.36)	3.32 (2.28 to 4.62)	36.69 (-5.76 to 105.97)	-1.65 (-1.93 to -1.36)
Palau	0 (0 to 1)	8.04 (6.05 to 10.31)	4.10 (2.89 to 5.65)	1 (0 to 1)	7.81 (6.23 to 9.94)	2.99 (2.12 to 4.19)	62.46 (10.79 to 143.85)	-1.03 (-1.07 to -0.99)
Palestine	102 (74 to 135)	10.54 (7.97 to 13.31)	13.97 (9.95 to 18.69)	144 (110 to 187)	10.00 (8.02 to 12.50)	7.29 (5.47 to 9.67)	41.10 (2.33 to 101.78)	-2.39 (-2.73 to -2.05)
Panama	32 (24 to 41)	6.51 (4.97 to 8.42)	2.34 (1.73 to 3.05)	41 (28 to 59)	3.83 (2.83 to 5.16)	0.91 (0.62 to 1.30)	29.81 (-7.95 to 74.47)	-3.45 (-3.74 to -3.15)
Papua New Guinea	34 (20 to 54)	7.30 (5.57 to 9.55)	2.14 (1.32 to 3.46)	74 (45 to 113)	6.21 (4.68 to 8.19)	1.70 (1.02 to 2.59)	121.52 (46.18 to 245.17)	-0.94 (-1.06 to -0.83)
Paraguay	166 (124 to 214)	15.03 (11.78 to 18.46)	8.48 (6.21 to 11.09)	207 (137 to 290)	10.41 (7.56 to 13.73)	3.94 (2.61 to 5.50)	24.65 (-11.92 to 76.27)	-2.37 (-2.59 to -2.14)
Peru	112 (80 to 149)	4.14 (3.06 to 5.36)	1.04 (0.74 to 1.39)	204 (134 to 291)	4.19 (3.13 to 5.60)	0.62 (0.40 to 0.89)	82.47 (23.67 to 158.03)	-2.36 (-2.88 to -1.83)
Philippines	1462 (1153 to 1831)	14.25 (11.66 to 17.57)	6.85 (5.08 to 8.87)	2870 (2226 to 3663)	10.16 (8.35 to 12.35)	3.92 (3.03 to 4.99)	96.36 (52.97 to 160.69)	-1.64 (-1.82 to -1.45)

Location	1990			2021			1990-2021	
	Deaths Cases (95%UI)	PAF, % (95%UI)	ASMR (95%UI)	Deaths Cases (95%UI)	PAF, % (95%UI)	ASMR (95%UI)	Cases change, % (95%UI)	EAPC (95%CI)
Poland	5335 (4433 to 6423)	10.82 (8.96 to 12.95)	12.48 (10.30 to 15.19)	2251 (1781 to 2868)	6.50 (5.18 to 8.04)	3.02 (2.41 to 3.81)	-57.80 (-63.40 to -50.91)	-4.98 (-5.13 to -4.84)
Portugal	1157 (918 to 1454)	5.88 (4.68 to 7.39)	8.31 (6.54 to 10.47)	224 (163 to 300)	2.35 (1.72 to 3.19)	0.80 (0.60 to 1.05)	-80.65 (-83.92 to -77.07)	-8.19 (-8.49 to -7.89)
Puerto Rico	54 (39 to 73)	5.46 (4.03 to 7.35)	1.58 (1.11 to 2.12)	37 (25 to 53)	4.10 (2.93 to 5.67)	0.46 (0.32 to 0.64)	-31.66 (-48.90 to -12.04)	-4.52 (-4.76 to -4.28)
Qatar	3 (2 to 4)	8.37 (6.68 to 10.30)	4.49 (3.16 to 6.08)	10 (6 to 14)	8.59 (6.55 to 10.58)	1.48 (0.96 to 2.11)	203.72 (101.74 to 364.79)	-4.06 (-4.81 to -3.31)
Republic of Korea	3151 (2496 to 3856)	14.66 (11.79 to 17.89)	14.46 (10.93 to 18.37)	2024 (1448 to 2723)	8.73 (6.49 to 11.41)	2.19 (1.55 to 2.95)	-35.75 (-51.72 to -18.51)	-6.89 (-7.19 to -6.6)
Republic of Moldova	177 (133 to 230)	6.42 (4.96 to 8.01)	4.35 (3.25 to 5.75)	282 (227 to 350)	9.02 (7.46 to 11.05)	4.60 (3.69 to 5.71)	59.86 (29.68 to 104.74)	0.6 (0.03 to 1.18)
Romania	2620 (2054 to 3289)	7.67 (6.07 to 9.51)	10.41 (7.91 to 13.33)	1735 (1336 to 2231)	4.54 (3.57 to 5.76)	4.42 (3.47 to 5.61)	-33.77 (-46.76 to -18.61)	-3.58 (-4 to -3.16)
Russian Federation	19672 (16876 to 22931)	7.03 (6.01 to 8.18)	11.02 (9.45 to 12.91)	17985 (14706 to 21761)	7.49 (6.28 to 9.08)	7.40 (6.08 to 8.89)	-8.57 (-20.95 to 3.10)	-2.05 (-2.87 to -1.23)
Rwanda	161 (110 to 234)	10.28 (7.71 to 13.02)	7.97 (5.26 to 11.64)	199 (123 to 298)	10.64 (7.71 to 14.15)	4.68 (2.79 to 7.07)	23.32 (-26.17 to 93.86)	-2.49 (-2.83 to -2.15)
Saint Kitts and Nevis	2 (1 to 2)	3.39 (2.53 to 4.45)	4.20 (3.03 to 5.70)	1 (1 to 1)	3.14 (2.35 to 4.22)	1.85 (1.29 to 2.55)	-33.74 (-50.16 to -12.66)	-2.77 (-2.93 to -2.6)
Saint Lucia	4 (3 to 5)	4.46 (3.49 to 5.64)	4.52 (3.44 to 5.96)	4 (3 to 5)	3.10 (2.33 to 4.10)	1.57 (1.10 to 2.12)	1.19 (-21.44 to 31.08)	-3.99 (-4.35 to -3.63)
Saint Vincent and the Grenadines	2 (1 to 2)	3.08 (2.36 to 4.03)	2.15 (1.60 to 2.83)	2 (2 to 3)	3.53 (2.65 to 4.64)	1.56 (1.13 to 2.11)	41.23 (15.35 to 71.77)	-0.96 (-1.14 to -0.79)

Location	1990			2021			1990-2021	
	Deaths Cases (95%UI)	PAF, % (95%UI)	ASMR (95%UI)	Deaths Cases (95%UI)	PAF, % (95%UI)	ASMR (95%UI)	Cases change, % (95%UI)	EAPC (95%CI)
Samoa	3 (2 to 4)	9.99 (7.90 to 12.34)	4.19 (3.00 to 5.65)	4 (3 to 6)	8.82 (6.69 to 11.22)	3.21 (2.32 to 4.32)	31.94 (-6.99 to 86.40)	-1.14 (-1.29 to -1)
San Marino	1 (1 to 2)	7.88 (5.62 to 10.43)	3.93 (2.77 to 5.40)	1 (1 to 1)	5.05 (3.40 to 6.94)	0.89 (0.55 to 1.38)	-42.07 (-62.89 to -10.74)	-4.14 (-4.48 to -3.8)
Sao Tome and Principe	1 (1 to 1)	2.19 (1.58 to 2.89)	1.26 (0.90 to 1.77)	2 (1 to 2)	2.50 (1.77 to 3.38)	1.41 (0.95 to 2.06)	92.09 (34.41 to 175.55)	0.19 (-0.18 to 0.57)
Saudi Arabia	211 (149 to 298)	4.99 (3.92 to 6.28)	3.89 (2.74 to 5.50)	638 (468 to 859)	8.61 (6.85 to 10.56)	3.10 (2.30 to 4.15)	202.68 (105.55 to 365.73)	-0.83 (-1 to -0.66)
Senegal	107 (77 to 143)	5.09 (3.84 to 6.45)	3.29 (2.38 to 4.46)	151 (101 to 217)	3.31 (2.41 to 4.45)	1.99 (1.32 to 2.86)	41.75 (-1.87 to 95.88)	-1.86 (-1.97 to -1.74)
Serbia	1653 (1267 to 2099)	9.14 (7.36 to 11.03)	17.59 (13.13 to 22.34)	1484 (1099 to 2019)	6.62 (5.16 to 8.32)	8.55 (6.37 to 11.59)	-10.23 (-33.31 to 18.33)	-2.78 (-3.26 to -2.3)
Seychelles	4 (3 to 6)	12.42 (9.73 to 15.26)	7.72 (5.53 to 9.94)	4 (3 to 6)	10.14 (7.89 to 12.63)	3.94 (2.77 to 5.30)	-4.88 (-24.09 to 19.33)	-2 (-2.25 to -1.75)
Sierra Leone	80 (55 to 110)	5.25 (3.92 to 6.69)	4.04 (2.85 to 5.55)	95 (60 to 143)	4.08 (2.94 to 5.47)	2.56 (1.67 to 3.79)	19.45 (-19.40 to 74.19)	-1.31 (-1.47 to -1.14)
Singapore	72 (56 to 88)	7.49 (5.88 to 9.41)	3.63 (2.75 to 4.62)	20 (15 to 27)	3.56 (2.68 to 4.71)	0.24 (0.18 to 0.32)	-71.84 (-77.88 to -64.19)	-8.56 (-8.92 to -8.21)
Slovakia	503 (392 to 635)	9.02 (7.21 to 11.12)	8.46 (6.62 to 10.77)	331 (243 to 448)	7.07 (5.52 to 8.90)	3.37 (2.47 to 4.56)	-34.23 (-51.14 to -13.16)	-2.9 (-3 to -2.8)
Slovenia	144 (114 to 180)	7.07 (5.69 to 8.80)	5.85 (4.63 to 7.33)	59 (43 to 81)	4.11 (3.01 to 5.41)	1.21 (0.88 to 1.62)	-58.82 (-67.00 to -50.18)	-4.86 (-5.07 to -4.66)
Solomon Islands	5 (3 to 7)	11.24 (8.82 to 13.95)	4.24 (2.86 to 6.46)	11 (7 to 17)	9.60 (7.37 to 11.94)	3.68 (2.49 to 5.76)	129.29 (61.59 to 238.96)	-0.31 (-0.64 to 0.03)

Location	1990			2021			1990-2021	
	Deaths Cases (95%UI)	PAF, % (95%UI)	ASMR (95%UI)	Deaths Cases (95%UI)	PAF, % (95%UI)	ASMR (95%UI)	Cases change, % (95%UI)	EAPC (95%CI)
Somalia	34 (16 to 64)	4.90 (2.95 to 7.66)	2.02 (0.98 to 3.81)	56 (28 to 105)	4.08 (2.40 to 6.50)	1.21 (0.61 to 2.21)	62.54 (5.45 to 167.19)	-1.67 (-1.81 to -1.53)
South Africa	762 (563 to 983)	10.47 (8.34 to 12.70)	4.18 (3.05 to 5.43)	870 (677 to 1095)	4.40 (3.48 to 5.40)	2.09 (1.59 to 2.66)	14.19 (-7.71 to 46.06)	-2.39 (-2.76 to -2.02)
South Sudan	54 (34 to 87)	5.43 (3.70 to 7.65)	2.43 (1.51 to 3.87)	48 (29 to 77)	4.49 (3.05 to 6.55)	1.65 (0.99 to 2.68)	-11.84 (-45.36 to 44.33)	-1.5 (-1.73 to -1.27)
Spain	3155 (2421 to 3909)	8.59 (6.58 to 10.54)	5.74 (4.38 to 7.12)	808 (580 to 1049)	4.09 (2.97 to 5.41)	0.69 (0.51 to 0.88)	-74.38 (-78.92 to -69.66)	-6.95 (-7.26 to -6.64)
Sri Lanka	669 (510 to 869)	9.23 (7.13 to 11.62)	8.64 (6.27 to 11.53)	639 (392 to 925)	4.20 (3.20 to 5.43)	2.74 (1.69 to 4.00)	-4.57 (-39.76 to 38.30)	-3.26 (-3.43 to -3.08)
Sudan	533 (345 to 771)	7.03 (5.12 to 9.39)	6.57 (4.31 to 9.35)	793 (530 to 1185)	6.57 (4.79 to 8.77)	4.76 (3.15 to 7.00)	48.62 (3.52 to 121.26)	-1.22 (-1.35 to -1.09)
Suriname	10 (8 to 13)	8.16 (6.58 to 10.07)	4.29 (3.34 to 5.37)	14 (10 to 20)	6.01 (4.61 to 7.80)	2.33 (1.55 to 3.28)	41.52 (-1.06 to 96.46)	-2.36 (-2.78 to -1.95)
Sweden	736 (558 to 960)	8.95 (6.72 to 11.60)	4.27 (3.27 to 5.53)	300 (202 to 414)	6.52 (4.52 to 8.70)	1.10 (0.76 to 1.50)	-59.22 (-69.15 to -49.11)	-4.49 (-4.79 to -4.2)
Switzerland	480 (347 to 630)	9.44 (7.01 to 12.29)	4.12 (3.00 to 5.39)	193 (129 to 265)	6.60 (4.53 to 8.92)	0.83 (0.57 to 1.11)	-59.66 (-69.09 to -48.43)	-4.96 (-5.16 to -4.77)
Syrian Arab Republic	424 (321 to 557)	12.21 (9.96 to 14.82)	9.19 (6.87 to 12.25)	603 (417 to 819)	9.79 (7.80 to 11.92)	5.54 (3.77 to 7.43)	42.15 (-3.31 to 114.44)	-2.19 (-2.4 to -1.97)
Taiwan (Province of China)	841 (689 to 999)	12.92 (10.60 to 15.12)	6.13 (4.92 to 7.43)	532 (405 to 670)	8.32 (6.42 to 10.60)	1.22 (0.94 to 1.53)	-36.75 (-49.61 to -23.41)	-5.16 (-5.41 to -4.91)
Tajikistan	204 (150 to 274)	10.27 (7.99 to 12.86)	7.93 (5.75 to 10.69)	167 (112 to 239)	6.05 (4.48 to 8.03)	3.59 (2.36 to 5.20)	-18.07 (-46.83 to 22.15)	-2.56 (-2.92 to -2.19)

Location	1990			2021			1990-2021	
	Deaths Cases (95%UI)	PAF, % (95%UI)	ASMR (95%UI)	Deaths Cases (95%UI)	PAF, % (95%UI)	ASMR (95%UI)	Cases change, % (95%UI)	EAPC (95%CI)
Thailand	1525 (1122 to 1993)	13.08 (10.38 to 16.06)	5.13 (3.72 to 6.76)	2326 (1654 to 3235)	8.12 (6.40 to 9.93)	2.14 (1.52 to 2.99)	52.49 (6.13 to 120.63)	-3.43 (-3.64 to -3.21)
Timor-Leste	12 (8 to 16)	11.19 (8.40 to 14.29)	6.14 (4.15 to 8.51)	45 (26 to 69)	9.90 (6.82 to 13.16)	6.33 (3.64 to 9.45)	287.93 (140.32 to 500.57)	0.23 (0.1 to 0.36)
Togo	63 (46 to 85)	8.85 (6.82 to 11.33)	6.02 (4.36 to 8.18)	139 (94 to 196)	6.94 (5.20 to 9.12)	4.04 (2.68 to 5.70)	120.12 (53.89 to 214.53)	-1.48 (-1.62 to -1.33)
Tokelau	0 (0 to 0)	6.84 (5.04 to 9.03)	3.73 (2.48 to 5.17)	0 (0 to 0)	5.23 (3.90 to 6.76)	2.22 (1.61 to 3.12)	-33.23 (-52.59 to -4.55)	-1.94 (-2.05 to -1.83)
Tonga	2 (1 to 2)	12.88 (10.05 to 15.84)	3.56 (2.54 to 4.94)	2 (1 to 3)	8.81 (6.60 to 11.33)	2.57 (1.83 to 3.49)	17.07 (-16.59 to 63.64)	-1.21 (-1.41 to -1.01)
Trinidad and Tobago	42 (33 to 53)	7.48 (6.04 to 9.32)	5.29 (4.14 to 6.69)	39 (28 to 54)	5.09 (3.92 to 6.53)	2.05 (1.43 to 2.84)	-6.54 (-30.37 to 23.61)	-3.52 (-3.82 to -3.21)
Tunisia	397 (276 to 543)	13.99 (10.76 to 17.63)	9.99 (6.75 to 13.82)	759 (512 to 1100)	11.22 (8.23 to 14.57)	6.33 (4.22 to 9.15)	91.04 (34.21 to 174.17)	-1.74 (-1.92 to -1.56)
Turkey	121 (85 to 155)	12.98 (10.48 to 15.69)	6.51 (4.66 to 8.32)	227 (138 to 320)	6.95 (5.48 to 8.71)	5.88 (3.74 to 8.32)	87.37 (34.93 to 153.06)	-0.52 (-1.08 to 0.04)
Turkmenistan	0 (0 to 0)	10.12 (8.06 to 12.32)	4.57 (3.18 to 6.29)	0 (0 to 0)	7.75 (5.84 to 9.60)	3.54 (2.53 to 4.84)	22.67 (-12.58 to 72.31)	-0.92 (-1 to -0.84)
Tuvalu	2859 (2170 to 3551)	9.45 (7.44 to 12.02)	9.30 (6.96 to 11.61)	2618 (1874 to 3564)	8.10 (6.23 to 10.35)	3.01 (2.15 to 4.11)	-8.41 (-31.80 to 22.71)	-4.07 (-4.37 to -3.78)
Uganda	90 (60 to 136)	3.93 (2.92 to 5.20)	1.83 (1.20 to 2.79)	123 (82 to 180)	3.25 (2.36 to 4.34)	1.10 (0.73 to 1.62)	36.80 (-12.44 to 105.30)	-2.75 (-3.22 to -2.28)
Ukraine	7943 (6337 to 9550)	8.08 (6.53 to 9.59)	11.24 (8.80 to 13.57)	4385 (2907 to 6061)	6.82 (4.79 to 9.04)	5.59 (3.72 to 7.72)	-44.80 (-62.17 to -26.17)	-2.95 (-3.46 to -2.44)

Location	1990			2021			1990-2021	
	Deaths Cases (95%UI)	PAF, % (95%UI)	ASMR (95%UI)	Deaths Cases (95%UI)	PAF, % (95%UI)	ASMR (95%UI)	Cases change, % (95%UI)	EAPC (95%CI)
United Arab Emirates	22 (15 to 31)	9.56 (7.72 to 11.69)	6.00 (4.19 to 8.38)	59 (41 to 80)	9.15 (7.21 to 11.11)	3.23 (2.21 to 4.51)	168.47 (95.51 to 273.51)	-0.71 (-1.25 to -0.17)
United Kingdom	7547 (5948 to 9431)	12.66 (9.95 to 15.61)	7.67 (6.09 to 9.58)	1587 (1144 to 2143)	6.32 (4.54 to 8.55)	1.02 (0.75 to 1.37)	-78.97 (-82.62 to -74.93)	-6.91 (-7.14 to -6.68)
United Republic of Tanzania	247 (158 to 375)	8.10 (6.04 to 10.62)	2.96 (1.90 to 4.47)	534 (327 to 837)	5.61 (4.13 to 7.60)	2.61 (1.67 to 4.09)	116.38 (20.15 to 265.86)	-0.82 (-0.98 to -0.65)
United States of America	9443 (7430 to 11881)	9.50 (7.52 to 11.69)	2.80 (2.21 to 3.51)	7318 (5412 to 9740)	6.34 (4.81 to 8.48)	1.16 (0.88 to 1.53)	-22.50 (-33.37 to -9.65)	-3.5 (-3.92 to -3.08)
United States Virgin Islands	1 (1 to 2)	4.99 (3.60 to 6.69)	1.69 (1.15 to 2.41)	1 (1 to 2)	4.07 (2.76 to 5.65)	0.74 (0.47 to 1.05)	11.11 (-27.67 to 64.89)	-2.66 (-2.83 to -2.48)
Uruguay	181 (145 to 223)	6.34 (5.17 to 7.73)	4.57 (3.66 to 5.60)	109 (83 to 141)	4.71 (3.57 to 6.05)	1.85 (1.43 to 2.36)	-39.89 (-49.91 to -29.55)	-3.23 (-3.48 to -2.97)
Uzbekistan	350 (273 to 430)	5.51 (4.40 to 6.78)	3.03 (2.35 to 3.75)	865 (663 to 1100)	7.28 (5.98 to 8.69)	3.50 (2.63 to 4.47)	147.52 (89.18 to 224.38)	-0.09 (-0.64 to 0.46)
Vanuatu	2 (1 to 3)	8.74 (7.07 to 10.80)	3.93 (2.69 to 5.51)	4 (2 to 5)	6.36 (5.02 to 8.08)	2.41 (1.64 to 3.34)	71.67 (23.12 to 141.22)	-1.97 (-2.14 to -1.8)
Venezuela (Bolivarian Republic of)	197 (148 to 249)	7.62 (5.81 to 9.45)	2.29 (1.69 to 2.95)	315 (220 to 445)	4.31 (3.29 to 5.59)	1.13 (0.79 to 1.58)	59.80 (14.41 to 131.40)	-2.71 (-2.97 to -2.44)
Viet Nam	3867 (2786 to 5259)	12.90 (10.27 to 16.10)	10.58 (7.57 to 14.56)	8699 (6074 to 11691)	11.68 (9.01 to 14.64)	9.75 (6.89 to 13.10)	124.95 (55.21 to 224.93)	-0.31 (-0.43 to -0.18)
Yemen	462 (314 to 666)	11.69 (9.22 to 14.50)	10.64 (7.27 to 15.22)	1208 (793 to 1708)	11.13 (8.78 to 13.80)	9.89 (6.42 to 14.10)	161.52 (77.50 to 289.70)	-0.42 (-0.49 to -0.35)
Zambia	67 (44 to 103)	6.68 (5.18 to 8.42)	3.22 (2.09 to 4.97)	135 (85 to 198)	4.92 (3.58 to 6.45)	2.75 (1.69 to 4.23)	101.27 (28.18 to 206.29)	-0.84 (-1 to -0.69)

Location	1990			2021			1990-2021	
	Deaths Cases (95%UI)	PAF, % (95%UI)	ASMR (95%UI)	Deaths Cases (95%UI)	PAF, % (95%UI)	ASMR (95%UI)	Cases change, % (95%UI)	EAPC (95%CI)
Zimbabwe	130 (93 to 178)	9.59 (7.44 to 12.32)	4.32 (2.99 to 6.20)	260 (184 to 350)	8.68 (6.53 to 11.14)	5.06 (3.47 to 6.97)	99.21 (33.62 to 192.43)	0.98 (0.45 to 1.52)

Supplementary Table 4. DALYs of Ischemic Stroke due to active smoking at the national level. DALYs = Disability-Adjusted Life Years. ASDR = Age-standardized DALYs rate (per 100000 population). EAPC = estimated annual percentage change. **95%UI = 95% Uncertainty Interval. 95%CI = 95% Confidence Interval.**

Location	1990		2021		1990-2021	
	DALYs Cases (95%UI)	ASMR (95%UI)	DALYs Cases (95%UI)	ASMR (95%UI)	Cases change, % (95%UI)	EAPC (95%CI)
Afghanistan	7001 (4445 to 10846)	102.64 (64.54 to 157.50)	12852 (8780 to 19570)	116.23 (79.92 to 175.28)	83.57 (24.41 to 169.92)	0.48 (0.07 to 0.88)
Albania	2755 (2078 to 3523)	146.89 (111.20 to 187.16)	4623 (3224 to 6257)	106.64 (74.99 to 142.62)	67.80 (17.40 to 132.80)	-0.64 (-0.91 to -0.37)
Algeria	20532 (14886 to 27035)	202.01 (147.55 to 268.20)	36054 (26527 to 49598)	114.97 (82.51 to 160.56)	75.60 (33.07 to 131.44)	-1.92 (-2.02 to -1.82)
American Samoa	22 (17 to 27)	87.91 (67.95 to 112.63)	31 (24 to 41)	63.28 (48.59 to 81.48)	45.48 (14.62 to 87.91)	-1.41 (-1.58 to -1.24)
Andorra	32 (23 to 42)	55.40 (40.12 to 74.01)	35 (25 to 47)	22.87 (16.25 to 30.43)	11.05 (-23.72 to 51.37)	-2.87 (-3.09 to -2.65)
Angola	2939 (2074 to 4101)	77.15 (53.55 to 107.91)	7223 (5146 to 9639)	61.22 (43.39 to 82.54)	145.80 (67.76 to 252.50)	-0.83 (-1.08 to -0.59)

Location	1990		2021		1990-2021	
	DALYs Cases (95%UI)	ASMR (95%UI)	DALYs Cases (95%UI)	ASMR (95%UI)	Cases change, % (95%UI)	EAPC (95%CI)
Antigua and Barbuda	27 (21 to 35)	50.04 (39.13 to 63.57)	30 (23 to 39)	28.00 (21.26 to 36.98)	9.56 (-9.33 to 33.01)	-2.17 (-2.48 to -1.86)
Argentina	30489 (24684 to 37050)	93.32 (75.76 to 113.36)	18407 (14798 to 22771)	33.49 (26.86 to 41.32)	-39.63 (-48.52 to -29.27)	-3.17 (-3.28 to -3.05)
Armenia	4943 (4064 to 5777)	179.87 (146.54 to 212.32)	6308 (5233 to 7509)	145.25 (121.30 to 173.24)	27.64 (10.08 to 50.42)	-1.48 (-1.78 to -1.18)
Australia	12798 (10414 to 15603)	64.54 (52.54 to 78.37)	6493 (4992 to 8592)	15.34 (12.14 to 20.00)	-49.27 (-56.83 to -40.91)	-4.78 (-4.94 to -4.63)
Austria	11279 (9263 to 13829)	96.97 (80.80 to 118.53)	5684 (4355 to 7298)	33.44 (25.95 to 42.39)	-49.61 (-57.29 to -42.35)	-3.57 (-3.76 to -3.38)
Azerbaijan	5784 (4244 to 7837)	111.19 (82.24 to 150.28)	9214 (6571 to 12472)	91.74 (65.25 to 123.66)	59.31 (10.18 to 133.85)	-0.42 (-0.79 to -0.06)
Bahamas	60 (46 to 78)	38.42 (29.21 to 50.35)	99 (71 to 131)	24.44 (17.31 to 32.71)	64.41 (19.98 to 121.72)	-1.39 (-1.54 to -1.25)
Bahrain	257 (200 to 318)	157.41 (120.49 to 202.84)	639 (494 to 815)	74.39 (55.96 to 97.18)	148.76 (89.23 to 215.65)	-2.99 (-3.31 to -2.67)
Bangladesh	73242 (50185 to 111063)	164.33 (112.43 to 247.07)	158714 (107190 to 231816)	120.66 (81.07 to 175.77)	116.70 (61.21 to 196.44)	-0.93 (-1.12 to -0.74)
Barbados	156 (121 to 199)	51.97 (40.85 to 65.91)	129 (91 to 174)	25.22 (17.81 to 33.95)	-17.22 (-38.82 to 12.29)	-2.91 (-3.16 to -2.66)
Belarus	34840 (27690 to 41587)	265.07 (209.30 to 316.59)	31944 (24685 to 39903)	198.43 (154.46 to 247.37)	-8.31 (-24.64 to 15.40)	-1.65 (-2.16 to -1.14)
Belgium	18100 (14724 to 22251)	116.96 (95.54 to 142.36)	6534 (5168 to 8254)	28.63 (23.11 to 35.79)	-63.90 (-68.58 to -57.46)	-4.52 (-4.66 to -4.38)

Location	1990		2021		1990-2021	
	DALYs Cases (95%UI)	ASMR (95%UI)	DALYs Cases (95%UI)	ASMR (95%UI)	Cases change, % (95%UI)	EAPC (95%CI)
Belize	33 (26 to 40)	35.54 (27.74 to 43.89)	80 (62 to 102)	26.92 (20.45 to 34.70)	143.01 (94.40 to 203.89)	-1.06 (-1.48 to -0.64)
Benin	1423 (1044 to 1927)	71.01 (51.81 to 95.90)	1968 (1376 to 2803)	37.14 (25.90 to 52.27)	38.33 (-3.84 to 97.75)	-2.26 (-2.4 to -2.12)
Bermuda	35 (26 to 45)	56.42 (41.30 to 71.57)	32 (24 to 44)	24.24 (18.23 to 32.72)	-8.54 (-30.43 to 24.17)	-2.59 (-2.89 to -2.28)
Bhutan	105 (62 to 158)	47.52 (27.37 to 73.26)	209 (139 to 297)	35.72 (23.91 to 50.49)	99.79 (37.56 to 204.96)	-0.95 (-1.04 to -0.87)
Bolivia (Plurinational State of)	1627 (1044 to 2183)	51.05 (33.20 to 68.32)	2091 (1401 to 3009)	23.10 (15.44 to 33.32)	28.47 (-10.21 to 84.16)	-2.29 (-2.63 to -1.95)
Bosnia and Herzegovina	12604 (10049 to 15573)	311.37 (247.69 to 383.08)	13928 (10289 to 17910)	226.21 (168.80 to 290.59)	10.51 (-19.36 to 46.60)	-1.13 (-1.41 to -0.85)
Botswana	864 (634 to 1150)	163.36 (118.15 to 214.80)	1258 (928 to 1704)	89.66 (66.29 to 121.57)	45.53 (5.22 to 106.73)	-2.12 (-2.3 to -1.94)
Brazil	197093 (168004 to 228556)	223.05 (187.02 to 263.67)	121131 (98075 to 149329)	48.16 (38.74 to 59.66)	-38.54 (-45.33 to -30.87)	-5.14 (-5.43 to -4.86)
Brunei Darussalam	212 (167 to 264)	214.00 (166.37 to 267.81)	196 (153 to 250)	56.88 (43.84 to 74.10)	-7.64 (-26.67 to 18.32)	-4.25 (-4.53 to -3.97)
Bulgaria	42711 (35825 to 50590)	345.03 (291.15 to 410.58)	33796 (26212 to 41752)	260.57 (205.33 to 322.38)	-20.87 (-33.10 to -5.07)	-0.86 (-1.03 to -0.69)
Burkina Faso	1463 (980 to 2140)	32.10 (21.60 to 47.49)	2733 (1811 to 3973)	27.02 (18.30 to 38.76)	86.87 (34.46 to 166.63)	-0.45 (-0.63 to -0.28)
Burundi	1764 (1217 to 2657)	76.41 (52.36 to 115.38)	1330 (919 to 1983)	26.40 (17.76 to 39.08)	-24.59 (-49.09 to 12.23)	-3.68 (-4.27 to -3.09)

Location	1990		2021		1990-2021	
	DALYs Cases (95%UI)	ASMR (95%UI)	DALYs Cases (95%UI)	ASMR (95%UI)	Cases change, % (95%UI)	EAPC (95%CI)
Cabo Verde	114 (83 to 154)	53.21 (38.95 to 71.14)	192 (141 to 259)	40.18 (29.78 to 54.01)	67.72 (20.32 to 138.42)	-1.41 (-1.79 to -1.03)
Cambodia	7503 (5805 to 10017)	183.72 (140.64 to 243.50)	17515 (12604 to 23244)	158.19 (112.71 to 211.68)	133.44 (70.86 to 210.97)	-0.76 (-0.94 to -0.59)
Cameroon	2731 (1934 to 3923)	56.80 (40.04 to 81.69)	6977 (4467 to 10346)	49.63 (31.83 to 73.94)	155.46 (67.93 to 271.94)	-0.42 (-1.02 to 0.19)
Canada	27416 (22238 to 33772)	84.31 (68.11 to 103.98)	19109 (14668 to 24774)	28.12 (21.80 to 36.06)	-30.30 (-39.93 to -19.41)	-3.9 (-4.15 to -3.65)
Central African Republic	717 (467 to 1092)	62.70 (41.72 to 95.50)	938 (599 to 1482)	40.78 (26.88 to 63.73)	30.80 (-8.86 to 83.66)	-1.56 (-1.76 to -1.36)
Chad	1900 (1244 to 2848)	66.86 (43.56 to 99.28)	3803 (2508 to 5751)	64.49 (41.67 to 98.14)	100.17 (35.15 to 211.24)	-0.41 (-0.65 to -0.16)
Chile	7772 (6447 to 9467)	75.15 (62.34 to 91.77)	6101 (4900 to 7658)	24.63 (19.85 to 30.88)	-21.50 (-30.92 to -11.07)	-3.5 (-3.58 to -3.42)
China	1974215 (1590831 to 2491808)	242.55 (193.99 to 305.22)	3916454 (3034617 to 5040372)	185.51 (142.74 to 237.21)	98.38 (45.53 to 165.58)	-0.69 (-0.82 to -0.57)
Colombia	9094 (7565 to 10945)	50.05 (41.15 to 60.37)	6917 (5234 to 8992)	12.54 (9.47 to 16.29)	-23.93 (-38.68 to -6.87)	-5.14 (-5.51 to -4.76)
Comoros	144 (96 to 211)	78.58 (52.19 to 114.98)	190 (126 to 272)	41.74 (26.64 to 59.65)	32.49 (-8.53 to 94.85)	-2.49 (-2.75 to -2.23)
Congo	678 (459 to 919)	63.84 (43.32 to 86.84)	1463 (1019 to 2018)	55.75 (39.09 to 77.58)	115.93 (56.73 to 193.05)	-0.5 (-0.77 to -0.22)
Cook Islands	10 (8 to 13)	80.73 (60.63 to 104.65)	12 (8 to 15)	46.50 (34.02 to 62.28)	10.72 (-13.98 to 43.06)	-1.95 (-2.1 to -1.81)

Location	1990		2021		1990-2021	
	DALYs Cases (95%UI)	ASMR (95%UI)	DALYs Cases (95%UI)	ASMR (95%UI)	Cases change, % (95%UI)	EAPC (95%CI)
Costa Rica	700 (570 to 841)	40.07 (32.44 to 48.86)	922 (738 to 1156)	16.84 (13.46 to 21.14)	31.84 (10.96 to 54.19)	-3.23 (-3.47 to -2.99)
Croatia	18427 (14995 to 22335)	321.81 (259.86 to 392.52)	8858 (6889 to 11275)	96.82 (76.75 to 121.91)	-51.93 (-59.90 to -42.13)	-3.69 (-3.81 to -3.57)
Cuba	10046 (8091 to 12218)	98.89 (79.67 to 120.72)	12456 (9881 to 15368)	64.39 (51.26 to 79.60)	24.00 (-0.70 to 52.24)	-1.39 (-1.52 to -1.25)
Cyprus	798 (604 to 1008)	120.45 (88.74 to 157.16)	677 (511 to 881)	33.37 (25.28 to 43.54)	-15.17 (-34.18 to 9.85)	-4.5 (-4.67 to -4.34)
Czechia	48548 (38952 to 59289)	347.19 (279.51 to 424.15)	13815 (10968 to 17325)	67.16 (53.30 to 83.81)	-71.54 (-76.60 to -66.33)	-5.51 (-5.71 to -5.31)
Côte d'Ivoire	4467 (3194 to 6077)	101.70 (73.95 to 137.77)	9425 (6310 to 13443)	74.48 (50.58 to 106.98)	111.00 (43.89 to 202.17)	-1.71 (-2.16 to -1.26)
Democratic People's Republic of Korea	29557 (21542 to 39594)	174.47 (128.02 to 232.32)	56812 (42304 to 76219)	166.58 (124.20 to 223.08)	92.21 (43.95 to 163.81)	-0.34 (-0.51 to -0.16)
Democratic Republic of the Congo	5197 (3725 to 7408)	32.80 (23.15 to 46.23)	8403 (5549 to 13622)	21.62 (13.84 to 34.35)	61.69 (13.69 to 133.97)	-1.38 (-1.67 to -1.09)
Denmark	14589 (11808 to 17612)	173.85 (142.79 to 208.57)	5128 (4051 to 6439)	41.62 (33.38 to 52.05)	-64.85 (-68.99 to -60.34)	-5.06 (-5.32 to -4.8)
Djibouti	120 (77 to 186)	96.18 (60.97 to 152.49)	510 (354 to 750)	84.60 (57.10 to 121.99)	326.78 (169.50 to 549.75)	-0.45 (-0.5 to -0.4)
Dominica	26 (20 to 34)	44.18 (34.13 to 56.30)	27 (20 to 37)	32.66 (24.13 to 44.09)	3.99 (-18.17 to 33.72)	-0.98 (-1.31 to -0.65)
Dominican Republic	3021 (2367 to 3792)	86.82 (66.19 to 111.04)	6719 (4766 to 9212)	67.94 (47.93 to 93.83)	122.44 (58.16 to 198.53)	-0.56 (-0.87 to -0.26)

Location	1990		2021		1990-2021	
	DALYs Cases (95%UI)	ASMR (95%UI)	DALYs Cases (95%UI)	ASMR (95%UI)	Cases change, % (95%UI)	EAPC (95%CI)
Ecuador	3046 (2450 to 3698)	56.75 (45.30 to 69.41)	2517 (1862 to 3298)	15.40 (11.41 to 20.24)	-17.37 (-34.62 to 5.59)	-4.05 (-4.38 to -3.72)
Egypt	75820 (51404 to 116812)	299.43 (203.95 to 447.23)	172961 (126049 to 238237)	277.70 (204.37 to 379.27)	128.12 (53.58 to 230.36)	0.08 (-0.15 to 0.31)
El Salvador	624 (481 to 809)	20.50 (15.74 to 26.48)	806 (576 to 1089)	13.19 (9.41 to 17.76)	29.06 (-2.35 to 73.08)	-1.56 (-1.89 to -1.23)
Equatorial Guinea	116 (76 to 179)	59.69 (39.10 to 91.79)	186 (114 to 278)	35.09 (21.24 to 53.90)	59.25 (-6.00 to 169.47)	-2.05 (-2.43 to -1.68)
Eritrea	417 (256 to 747)	31.82 (19.16 to 55.71)	674 (451 to 1023)	21.31 (14.27 to 31.94)	61.64 (4.53 to 153.87)	-1.62 (-1.82 to -1.43)
Estonia	5659 (4692 to 6813)	275.00 (230.17 to 329.73)	1543 (1273 to 1912)	65.35 (54.26 to 80.29)	-72.72 (-76.98 to -68.31)	-5.88 (-6.46 to -5.3)
Eswatini	178 (124 to 242)	72.40 (49.45 to 99.81)	257 (165 to 384)	51.73 (33.17 to 77.04)	44.68 (-8.14 to 124.14)	-0.84 (-1.22 to -0.45)
Ethiopia	3251 (1998 to 5604)	15.62 (9.48 to 27.02)	4125 (2902 to 6018)	9.55 (6.54 to 14.10)	26.86 (-23.99 to 87.05)	-1.43 (-1.71 to -1.15)
Fiji	377 (296 to 470)	96.03 (74.70 to 120.88)	511 (389 to 663)	61.99 (47.49 to 80.81)	35.59 (6.83 to 74.68)	-1.73 (-1.92 to -1.53)
Finland	6793 (5565 to 8262)	97.51 (80.62 to 117.95)	3190 (2449 to 4170)	27.64 (21.76 to 35.72)	-53.03 (-60.05 to -45.65)	-3.91 (-3.97 to -3.86)
France	60193 (48082 to 73633)	71.52 (58.01 to 86.50)	31348 (24683 to 39860)	24.20 (19.39 to 30.09)	-47.92 (-54.76 to -40.44)	-3.42 (-3.55 to -3.29)
Gabon	213 (154 to 297)	37.10 (26.82 to 51.39)	390 (273 to 536)	35.91 (24.72 to 49.17)	83.34 (28.04 to 158.19)	-0.12 (-0.19 to -0.04)

Location	1990		2021		1990-2021	
	DALYs Cases (95%UI)	ASMR (95%UI)	DALYs Cases (95%UI)	ASMR (95%UI)	Cases change, % (95%UI)	EAPC (95%CI)
Gambia	525 (373 to 728)	138.99 (98.02 to 193.86)	914 (630 to 1285)	86.77 (58.78 to 121.20)	74.08 (17.87 to 146.28)	-1.81 (-1.99 to -1.63)
Georgia	8263 (5812 to 10776)	131.98 (92.73 to 173.37)	12720 (10523 to 15379)	217.07 (181.27 to 261.32)	53.94 (21.51 to 111.66)	2.07 (1.73 to 2.42)
Germany	177348 (142495 to 218912)	139.07 (112.53 to 169.62)	71441 (56038 to 90987)	40.25 (32.17 to 50.23)	-59.72 (-65.30 to -53.61)	-4.03 (-4.33 to -3.74)
Ghana	4954 (3624 to 6756)	79.27 (58.35 to 105.31)	14975 (10758 to 20265)	87.04 (62.90 to 115.06)	202.27 (108.96 to 327.59)	1.08 (0.8 to 1.36)
Greece	28202 (22867 to 34105)	187.11 (152.46 to 227.12)	13050 (9945 to 16781)	51.41 (40.65 to 63.55)	-53.73 (-60.40 to -45.95)	-4.69 (-4.9 to -4.48)
Greenland	83 (66 to 101)	257.59 (202.56 to 320.40)	54 (42 to 70)	79.71 (60.90 to 105.23)	-35.41 (-49.75 to -18.38)	-4.03 (-4.17 to -3.9)
Grenada	64 (50 to 81)	92.23 (71.77 to 116.46)	38 (28 to 48)	32.28 (24.51 to 41.45)	-41.19 (-52.57 to -24.61)	-3.47 (-3.63 to -3.31)
Guam	55 (43 to 69)	61.92 (49.30 to 78.48)	90 (70 to 113)	44.44 (34.64 to 55.54)	64.67 (30.34 to 110.14)	-1.04 (-1.24 to -0.83)
Guatemala	874 (679 to 1102)	26.96 (20.39 to 34.29)	1383 (1024 to 1812)	12.88 (9.48 to 17.13)	58.30 (17.25 to 111.17)	-3.03 (-3.32 to -2.74)
Guinea	2348 (1660 to 3200)	70.52 (50.04 to 95.84)	4414 (2916 to 6194)	75.80 (50.68 to 105.50)	88.00 (30.01 to 187.14)	0.45 (0.25 to 0.65)
Guinea-Bissau	274 (187 to 398)	64.47 (44.05 to 92.71)	568 (385 to 818)	67.14 (46.37 to 94.60)	107.32 (41.53 to 201.36)	0.62 (0.38 to 0.86)
Guyana	570 (463 to 715)	144.84 (117.09 to 183.18)	418 (292 to 570)	63.68 (44.60 to 87.08)	-26.65 (-46.26 to 1.13)	-2.07 (-2.26 to -1.87)

Location	1990		2021		1990-2021	
	DALYs Cases (95%UI)	ASMR (95%UI)	DALYs Cases (95%UI)	ASMR (95%UI)	Cases change, % (95%UI)	EAPC (95%CI)
Haiti	2348 (1635 to 3153)	69.76 (49.15 to 95.10)	2777 (1759 to 4213)	38.04 (23.99 to 57.44)	18.25 (-17.44 to 74.11)	-1.86 (-2.09 to -1.63)
Honduras	1269 (891 to 1712)	61.58 (42.96 to 83.06)	3672 (2446 to 4912)	59.59 (40.20 to 80.04)	189.40 (106.94 to 308.28)	0.17 (-0.07 to 0.42)
Hungary	52160 (44673 to 60752)	359.54 (309.50 to 418.98)	16744 (13507 to 20769)	95.05 (76.84 to 117.48)	-67.90 (-72.22 to -62.90)	-4.71 (-4.9 to -4.52)
Iceland	284 (225 to 354)	97.50 (77.62 to 120.70)	125 (95 to 166)	21.38 (16.38 to 28.34)	-56.17 (-63.92 to -46.97)	-5.13 (-5.29 to -4.96)
India	344852 (253944 to 487657)	78.45 (57.37 to 109.26)	575207 (434096 to 837979)	49.71 (37.20 to 71.13)	66.80 (31.60 to 106.49)	-1.64 (-1.73 to -1.54)
Indonesia	137915 (105854 to 172386)	144.89 (110.27 to 182.86)	455394 (295556 to 628787)	197.63 (131.82 to 270.38)	230.20 (138.60 to 339.51)	1.13 (0.91 to 1.35)
Iran (Islamic Republic of)	42819 (35104 to 52582)	151.40 (122.56 to 185.90)	65727 (53711 to 80076)	79.12 (63.83 to 96.82)	53.50 (30.03 to 80.25)	-1.99 (-2.1 to -1.88)
Iraq	33672 (26076 to 41818)	429.38 (328.61 to 530.80)	65035 (47391 to 85208)	283.38 (206.92 to 373.31)	93.14 (36.29 to 164.27)	-1.94 (-2.12 to -1.76)
Ireland	6869 (5607 to 8325)	165.13 (134.43 to 200.99)	1639 (1240 to 2109)	20.44 (15.65 to 25.91)	-76.13 (-79.78 to -71.85)	-7.13 (-7.41 to -6.85)
Israel	3590 (2871 to 4374)	74.39 (59.56 to 89.93)	2326 (1802 to 3029)	19.20 (14.99 to 24.67)	-35.20 (-44.55 to -24.35)	-4.85 (-5.03 to -4.67)
Italy	100964 (82033 to 122564)	113.25 (92.11 to 136.87)	32642 (24903 to 41885)	22.24 (17.78 to 28.27)	-67.67 (-72.33 to -62.79)	-5.54 (-5.89 to -5.19)
Jamaica	1464 (1174 to 1809)	81.62 (65.28 to 100.31)	1364 (997 to 1906)	44.34 (32.51 to 61.88)	-6.85 (-30.46 to 22.98)	-1.91 (-2.35 to -1.47)

Location	1990		2021		1990-2021	
	DALYs Cases (95%UI)	ASMR (95%UI)	DALYs Cases (95%UI)	ASMR (95%UI)	Cases change, % (95%UI)	EAPC (95%CI)
Japan	217029 (181477 to 260443)	129.36 (107.45 to 156.01)	111533 (86370 to 142669)	34.95 (27.89 to 43.94)	-48.61 (-55.44 to -41.60)	-4.63 (-4.8 to -4.45)
Jordan	3952 (2970 to 5087)	300.80 (227.31 to 386.78)	10167 (7894 to 12606)	133.49 (101.91 to 168.33)	157.25 (95.13 to 236.52)	-3.05 (-3.26 to -2.84)
Kazakhstan	31595 (25533 to 37552)	234.43 (188.46 to 278.91)	28373 (23129 to 33980)	148.61 (120.51 to 178.87)	-10.20 (-26.63 to 9.00)	-2.09 (-2.82 to -1.36)
Kenya	3079 (2202 to 4214)	39.81 (27.90 to 55.33)	6065 (4512 to 8195)	27.19 (19.70 to 36.91)	96.98 (50.50 to 156.45)	-1.58 (-1.71 to -1.46)
Kiribati	77 (62 to 94)	197.75 (158.72 to 243.58)	164 (124 to 213)	211.98 (161.06 to 275.43)	113.20 (67.59 to 189.32)	0.06 (-0.16 to 0.28)
Kuwait	668 (551 to 806)	91.41 (74.89 to 109.73)	2302 (1798 to 2872)	64.93 (49.55 to 84.29)	244.74 (182.42 to 315.92)	-0.92 (-1.77 to -0.05)
Kyrgyzstan	6748 (5444 to 8180)	223.31 (179.21 to 271.43)	11796 (9449 to 14450)	225.13 (178.82 to 276.86)	74.80 (40.35 to 122.36)	0.05 (-0.31 to 0.4)
Lao People's Democratic Republic	5359 (3899 to 7077)	264.49 (194.71 to 342.22)	8187 (5807 to 11036)	186.04 (131.76 to 247.75)	52.78 (7.00 to 116.19)	-1.14 (-1.21 to -1.06)
Latvia	10058 (8394 to 11991)	280.44 (233.96 to 334.35)	5137 (4094 to 6420)	141.61 (114.85 to 175.63)	-48.93 (-56.97 to -39.24)	-2.6 (-2.87 to -2.32)
Lebanon	4624 (3447 to 6264)	213.46 (158.10 to 287.49)	5799 (4475 to 7291)	95.09 (74.03 to 118.66)	25.42 (-9.57 to 69.85)	-2.73 (-2.92 to -2.53)
Lesotho	605 (414 to 846)	77.98 (53.68 to 110.37)	1399 (885 to 2167)	140.02 (88.50 to 214.74)	131.29 (46.78 to 260.54)	2.7 (2.36 to 3.04)
Liberia	698 (513 to 933)	59.08 (43.10 to 78.35)	1112 (736 to 1551)	45.01 (30.09 to 63.01)	59.36 (12.16 to 134.07)	-1.14 (-1.3 to -0.98)

Location	1990		2021		1990-2021	
	**DALYs Cases (95%UI)**	**ASMR (95%UI)**	**DALYs Cases (95%UI)**	**ASMR (95%UI)**	**Cases change, % (95%UI)**	**EAPC (95%CI)**
Libya	1749 (1256 to 2397)	89.99 (63.63 to 125.20)	5877 (3985 to 8317)	100.15 (68.55 to 141.92)	235.93 (139.70 to 387.44)	0.78 (0.59 to 0.97)
Lithuania	7953 (6731 to 9420)	177.34 (149.93 to 210.31)	5495 (4443 to 6806)	105.66 (86.63 to 129.57)	-30.90 (-41.85 to -19.61)	-1.5 (-1.83 to -1.18)
Luxembourg	803 (630 to 1009)	146.32 (114.37 to 182.76)	252 (189 to 336)	23.73 (18.08 to 31.10)	-68.68 (-74.37 to -61.53)	-6.05 (-6.23 to -5.86)
Madagascar	4155 (3095 to 5630)	86.51 (63.15 to 118.99)	4178 (2873 to 5938)	36.87 (24.79 to 52.42)	0.55 (-30.02 to 40.49)	-2.97 (-3.32 to -2.62)
Malawi	2659 (1935 to 3575)	77.63 (54.99 to 105.52)	6016 (3926 to 8821)	85.20 (56.03 to 124.65)	126.30 (64.60 to 211.25)	0.06 (-0.21 to 0.32)
Malaysia	12377 (9938 to 15584)	134.18 (106.64 to 168.48)	21513 (17476 to 26379)	75.73 (61.24 to 93.14)	73.82 (38.38 to 113.72)	-1.8 (-1.9 to -1.71)
Maldives	206 (164 to 256)	264.82 (201.57 to 333.29)	294 (227 to 372)	89.38 (68.19 to 114.72)	42.32 (9.16 to 84.96)	-3.98 (-4.17 to -3.79)
Mali	1277 (851 to 1894)	33.62 (22.07 to 48.34)	4224 (2921 to 5948)	50.15 (34.87 to 70.79)	230.78 (133.77 to 372.72)	1.86 (1.6 to 2.11)
Malta	463 (376 to 561)	107.90 (87.50 to 131.81)	169 (129 to 220)	18.83 (14.97 to 23.92)	-63.60 (-69.75 to -56.09)	-5.82 (-5.97 to -5.68)
Marshall Islands	15 (10 to 21)	83.78 (58.26 to 120.71)	29 (20 to 43)	75.60 (51.69 to 111.64)	98.41 (46.64 to 166.43)	-0.29 (-0.34 to -0.25)
Mauritania	899 (640 to 1296)	83.89 (59.97 to 120.89)	1056 (681 to 1599)	44.81 (29.07 to 67.60)	17.44 (-13.97 to 61.10)	-2.27 (-2.52 to -2.03)
Mauritius	2067 (1771 to 2416)	271.15 (230.85 to 317.14)	1519 (1267 to 1821)	83.26 (69.45 to 99.38)	-26.49 (-36.47 to -15.94)	-4.8 (-5.39 to -4.21)

Location	1990		2021		1990-2021	
	DALYs Cases (95%UI)	ASMR (95%UI)	DALYs Cases (95%UI)	ASMR (95%UI)	Cases change, % (95%UI)	EAPC (95%CI)
Mexico	26695 (22355 to 31692)	66.05 (54.16 to 79.32)	20909 (16801 to 26048)	16.53 (13.11 to 20.83)	-21.68 (-34.35 to -7.07)	-4.82 (-4.98 to -4.66)
Micronesia (Federated States of)	79 (57 to 110)	151.97 (108.48 to 212.06)	104 (75 to 145)	124.47 (88.92 to 173.90)	31.63 (-4.44 to 79.83)	-0.74 (-0.8 to -0.67)
Monaco	95 (69 to 129)	134.54 (99.69 to 179.81)	44 (31 to 62)	46.63 (34.30 to 63.80)	-53.55 (-65.86 to -35.76)	-3.6 (-3.76 to -3.43)
Mongolia	672 (504 to 859)	61.66 (46.00 to 79.16)	2061 (1481 to 2727)	79.62 (55.67 to 105.62)	206.81 (115.95 to 325.56)	1.16 (0.79 to 1.53)
Montenegro	644 (496 to 844)	104.17 (80.27 to 137.04)	1046 (762 to 1411)	106.18 (78.52 to 142.72)	62.36 (17.66 to 122.31)	0.14 (-0.16 to 0.44)
Morocco	18030 (12531 to 24796)	123.04 (85.67 to 167.09)	28485 (19594 to 38652)	79.43 (54.45 to 108.20)	57.98 (15.41 to 114.53)	-1.44 (-1.6 to -1.28)
Mozambique	4554 (3404 to 5949)	79.04 (58.41 to 104.26)	10972 (6992 to 15476)	95.61 (62.26 to 135.11)	140.92 (63.14 to 238.89)	1.2 (0.99 to 1.41)
Myanmar	77351 (55917 to 100106)	354.31 (257.09 to 457.87)	61790 (44521 to 80660)	132.72 (94.53 to 174.78)	-20.12 (-41.51 to 11.68)	-3.4 (-3.51 to -3.3)
Namibia	905 (690 to 1170)	166.32 (125.09 to 220.71)	1189 (863 to 1561)	102.00 (72.17 to 135.57)	31.31 (-3.50 to 78.26)	-1.97 (-2.27 to -1.67)
Nauru	12 (8 to 16)	228.74 (156.06 to 306.26)	12 (9 to 16)	179.64 (129.92 to 239.29)	2.88 (-22.66 to 41.18)	-1.01 (-1.33 to -0.68)
Nepal	14774 (9928 to 20819)	174.94 (118.56 to 244.73)	19159 (13427 to 27994)	88.51 (61.31 to 127.13)	29.68 (-4.81 to 77.55)	-2.47 (-2.62 to -2.31)
Netherlands	22857 (18264 to 28150)	113.50 (91.40 to 139.27)	12431 (9677 to 16112)	35.16 (27.83 to 45.12)	-45.62 (-53.09 to -36.61)	-3.98 (-4.16 to -3.81)

Location	1990		2021		1990-2021	
	DALYs Cases (95%UI)	ASMR (95%UI)	DALYs Cases (95%UI)	ASMR (95%UI)	Cases change, % (95%UI)	EAPC (95%CI)
New Zealand	3228 (2631 to 3985)	81.04 (66.03 to 100.22)	2041 (1548 to 2653)	24.27 (18.71 to 31.56)	-36.75 (-45.59 to -26.79)	-4.02 (-4.31 to -3.73)
Nicaragua	572 (449 to 717)	37.25 (29.22 to 47.56)	923 (697 to 1242)	18.76 (14.05 to 25.65)	61.50 (22.11 to 114.48)	-2.02 (-2.19 to -1.84)
Niger	705 (474 to 1106)	26.30 (17.38 to 40.06)	1785 (1162 to 2826)	23.74 (15.39 to 35.90)	153.24 (78.86 to 259.64)	-0.31 (-0.36 to -0.25)
Nigeria	17384 (12062 to 25342)	39.91 (28.17 to 56.82)	24116 (16526 to 34410)	25.04 (17.12 to 35.43)	38.73 (-14.11 to 131.42)	-1.64 (-1.93 to -1.35)
Niue	2 (1 to 3)	90.43 (68.81 to 120.30)	2 (1 to 2)	71.79 (53.22 to 96.46)	-18.75 (-38.74 to 10.28)	-1.04 (-1.15 to -0.93)
North Macedonia	8566 (6908 to 10696)	473.31 (375.94 to 596.03)	10069 (7567 to 13107)	323.33 (240.27 to 425.26)	17.55 (-12.11 to 56.11)	-1.5 (-1.86 to -1.15)
Northern Mariana Islands	19 (14 to 24)	89.72 (67.74 to 117.18)	31 (24 to 39)	56.75 (43.69 to 72.76)	64.53 (21.08 to 120.69)	-1.84 (-2.05 to -1.63)
Norway	8461 (6747 to 10387)	121.16 (97.99 to 145.91)	2090 (1553 to 2739)	21.45 (16.34 to 27.87)	-75.30 (-79.57 to -70.28)	-6.15 (-6.37 to -5.94)
Oman	938 (684 to 1229)	123.33 (88.82 to 163.04)	1495 (1097 to 1901)	58.81 (43.34 to 77.05)	59.34 (14.16 to 129.72)	-1.87 (-2.1 to -1.63)
Pakistan	60986 (39552 to 88794)	112.41 (72.80 to 164.84)	90872 (66725 to 127820)	77.11 (56.34 to 106.42)	49.00 (6.65 to 109.08)	-1.55 (-1.83 to -1.27)
Palau	12 (9 to 15)	112.98 (82.46 to 149.64)	21 (16 to 28)	89.19 (66.94 to 118.51)	81.43 (33.26 to 151.58)	-0.82 (-0.86 to -0.77)
Palestine	2210 (1610 to 2862)	269.86 (194.96 to 350.68)	3636 (2844 to 4523)	148.84 (115.09 to 192.11)	64.53 (20.64 to 129.28)	-2.18 (-2.44 to -1.92)

Location	1990		2021		1990-2021	
	DALYs Cases (95%UI)	ASMR (95%UI)	DALYs Cases (95%UI)	ASMR (95%UI)	Cases change, % (95%UI)	EAPC (95%CI)
Panama	679 (535 to 848)	46.70 (36.19 to 58.84)	807 (588 to 1100)	18.19 (13.26 to 24.73)	18.92 (-10.32 to 51.96)	-3.4 (-3.69 to -3.11)
Papua New Guinea	1209 (803 to 1795)	61.28 (40.10 to 91.80)	2816 (1874 to 4024)	49.58 (32.80 to 70.32)	132.83 (62.76 to 246.17)	-0.88 (-1 to -0.77)
Paraguay	3166 (2484 to 3970)	151.92 (118.44 to 191.63)	4072 (2804 to 5644)	73.54 (49.99 to 101.66)	28.60 (-7.71 to 77.32)	-2.4 (-2.58 to -2.22)
Peru	2587 (1952 to 3340)	22.12 (16.58 to 28.71)	4833 (3419 to 6591)	14.33 (10.13 to 19.59)	86.80 (32.09 to 155.84)	-2.03 (-2.5 to -1.55)
Philippines	40133 (32631 to 48979)	144.87 (115.63 to 179.73)	86312 (69054 to 108053)	101.87 (81.39 to 126.41)	115.07 (75.89 to 173.21)	-1.06 (-1.23 to -0.88)
Poland	127913 (108866 to 148225)	292.31 (248.82 to 339.08)	56654 (46362 to 69810)	82.29 (68.12 to 100.66)	-55.71 (-61.03 to -49.56)	-4.37 (-4.5 to -4.25)
Portugal	24703 (20480 to 29750)	175.33 (145.86 to 210.43)	4885 (3857 to 6162)	21.97 (17.76 to 27.33)	-80.23 (-82.87 to -77.51)	-7.24 (-7.51 to -6.96)
Puerto Rico	1183 (890 to 1548)	33.04 (24.78 to 43.11)	842 (606 to 1156)	12.77 (9.48 to 17.18)	-28.80 (-44.11 to -10.04)	-3.53 (-3.73 to -3.34)
Qatar	140 (107 to 179)	106.78 (78.89 to 139.75)	612 (443 to 793)	38.53 (27.12 to 51.57)	336.49 (236.20 to 492.47)	-3.46 (-4 to -2.91)
Republic of Korea	83605 (68125 to 101489)	307.49 (247.30 to 372.40)	49204 (38574 to 62565)	53.37 (41.96 to 67.70)	-41.15 (-52.42 to -29.62)	-6.38 (-6.64 to -6.11)
Republic of Moldova	4959 (3872 to 6254)	111.92 (87.26 to 141.16)	7823 (6442 to 9563)	129.86 (107.32 to 158.46)	57.77 (29.90 to 98.20)	0.89 (0.33 to 1.45)
Romania	66017 (52974 to 80108)	240.65 (192.60 to 293.08)	41488 (33257 to 51095)	116.47 (93.96 to 142.22)	-37.16 (-47.91 to -23.75)	-3.16 (-3.54 to -2.77)

Location	1990		2021		1990-2021	
	DALYs Cases (95%UI)	ASMR (95%UI)	DALYs Cases (95%UI)	ASMR (95%UI)	Cases change, % (95%UI)	EAPC (95%CI)
Russian Federation	532293 (463620 to 608539)	288.52 (251.55 to 331.45)	484061 (404604 to 574477)	204.97 (171.48 to 242.55)	-9.06 (-19.64 to 0.80)	-1.88 (-2.63 to -1.11)
Rwanda	4069 (2886 to 5781)	163.82 (113.41 to 230.69)	4794 (3204 to 6854)	91.02 (58.37 to 132.74)	17.81 (-24.46 to 74.23)	-2.68 (-3.01 to -2.34)
Saint Kitts and Nevis	34 (25 to 44)	88.92 (67.52 to 114.92)	26 (19 to 36)	39.02 (28.21 to 53.09)	-21.52 (-40.59 to 3.78)	-2.87 (-3.06 to -2.68)
Saint Lucia	84 (67 to 103)	97.97 (77.14 to 121.17)	81 (61 to 105)	33.75 (25.41 to 43.81)	-4.06 (-24.80 to 24.28)	-3.82 (-4.09 to -3.54)
Saint Vincent and the Grenadines	34 (28 to 44)	48.08 (38.31 to 60.43)	49 (38 to 63)	34.44 (26.47 to 44.46)	41.45 (16.57 to 71.37)	-1.09 (-1.22 to -0.96)
Samoa	101 (78 to 130)	116.59 (90.43 to 149.68)	139 (107 to 180)	94.48 (72.50 to 122.14)	38.30 (5.18 to 84.29)	-0.93 (-1.05 to -0.8)
San Marino	30 (23 to 39)	84.48 (64.37 to 110.01)	19 (13 to 26)	25.41 (17.80 to 34.70)	-38.05 (-55.85 to -13.88)	-3.62 (-3.85 to -3.39)
Sao Tome and Principe	25 (18 to 33)	36.83 (26.69 to 49.68)	52 (35 to 74)	41.08 (27.85 to 58.41)	111.29 (50.84 to 197.49)	0.12 (-0.25 to 0.48)
Saudi Arabia	6523 (4662 to 9014)	98.92 (70.76 to 138.42)	25039 (18607 to 33505)	87.15 (66.36 to 113.54)	283.83 (161.61 to 479.78)	-0.38 (-0.55 to -0.21)
Senegal	3443 (2578 to 4580)	97.46 (72.89 to 129.71)	4722 (3258 to 6402)	55.72 (38.21 to 76.65)	37.15 (-2.11 to 86.53)	-2.06 (-2.16 to -1.95)
Serbia	40396 (32134 to 50381)	377.04 (294.68 to 469.57)	32597 (24982 to 42745)	198.92 (154.28 to 258.97)	-19.31 (-38.61 to 3.90)	-2.53 (-2.98 to -2.08)
Seychelles	106 (79 to 133)	187.08 (140.84 to 235.02)	116 (86 to 149)	98.95 (73.57 to 128.92)	9.55 (-8.99 to 35.76)	-1.96 (-2.16 to -1.76)

Location	1990		2021		1990-2021	
	DALYs Cases (95%UI)	ASMR (95%UI)	DALYs Cases (95%UI)	ASMR (95%UI)	Cases change, % (95%UI)	EAPC (95%CI)
Sierra Leone	2370 (1664 to 3151)	111.29 (78.79 to 148.39)	3114 (2006 to 4500)	73.38 (47.99 to 105.69)	31.40 (-7.67 to 86.52)	-1.15 (-1.31 to -0.98)
Singapore	2266 (1826 to 2733)	101.25 (80.58 to 124.46)	1205 (924 to 1602)	13.70 (10.50 to 18.16)	-46.81 (-56.14 to -37.27)	-6.53 (-6.65 to -6.4)
Slovakia	13277 (10763 to 16476)	222.03 (180.60 to 275.36)	9097 (6933 to 11765)	96.16 (74.28 to 123.84)	-31.48 (-47.39 to -12.73)	-2.6 (-2.69 to -2.52)
Slovenia	3619 (2949 to 4381)	147.51 (119.65 to 177.98)	1490 (1164 to 1919)	35.68 (28.29 to 45.15)	-58.83 (-64.78 to -51.33)	-4.38 (-4.59 to -4.17)
Solomon Islands	169 (122 to 240)	120.91 (86.75 to 171.68)	410 (297 to 586)	111.78 (80.84 to 160.79)	141.93 (81.99 to 233.98)	-0.09 (-0.39 to 0.22)
Somalia	1124 (614 to 1953)	48.90 (26.13 to 86.08)	1919 (1092 to 3390)	30.75 (16.95 to 53.61)	70.74 (17.40 to 158.01)	-1.55 (-1.69 to -1.42)
South Africa	22271 (17429 to 27238)	106.21 (81.05 to 131.49)	25151 (20356 to 30694)	53.03 (42.69 to 65.17)	12.93 (-4.62 to 35.68)	-2.33 (-2.6 to -2.06)
South Sudan	1447 (949 to 2281)	57.47 (37.66 to 90.04)	1403 (919 to 2190)	38.23 (24.45 to 59.77)	-3.00 (-36.73 to 48.95)	-1.56 (-1.8 to -1.31)
Spain	61689 (49744 to 74089)	113.17 (92.05 to 135.67)	22247 (17661 to 27795)	24.28 (19.69 to 29.83)	-63.94 (-68.59 to -58.56)	-4.95 (-5.23 to -4.67)
Sri Lanka	15095 (12090 to 18862)	158.72 (123.51 to 202.59)	13862 (9284 to 19693)	54.20 (36.39 to 76.90)	-8.17 (-38.59 to 27.92)	-3.29 (-3.4 to -3.18)
Sudan	14084 (9512 to 19884)	150.84 (100.12 to 212.72)	21925 (14766 to 32200)	108.30 (74.06 to 156.67)	55.68 (10.83 to 126.29)	-1.25 (-1.39 to -1.1)
Suriname	262 (214 to 317)	102.26 (83.07 to 124.12)	378 (263 to 518)	58.50 (40.64 to 80.56)	44.40 (2.20 to 95.66)	-2.23 (-2.62 to -1.84)

Location	1990		2021		1990-2021	
	**DALYs Cases (95%UI)**	**ASMR (95%UI)**	**DALYs Cases (95%UI)**	**ASMR (95%UI)**	**Cases change, % (95%UI)**	**EAPC (95%CI)**
Sweden	14421 (11221 to 18254)	91.55 (73.80 to 114.11)	6476 (4815 to 8571)	29.44 (22.41 to 38.32)	-55.09 (-63.44 to -46.42)	-3.89 (-4.06 to -3.73)
Switzerland	8712 (6848 to 10887)	80.37 (64.45 to 99.09)	3979 (3028 to 5142)	21.21 (16.46 to 26.89)	-54.33 (-62.34 to -45.91)	-4.22 (-4.44 to -4.01)
Syrian Arab Republic	11973 (9198 to 15356)	220.64 (169.87 to 285.96)	16678 (11892 to 22223)	125.13 (89.56 to 165.75)	39.30 (-2.34 to 105.11)	-2.35 (-2.57 to -2.13)
Taiwan (Province of China)	24112 (20199 to 28413)	154.32 (128.83 to 182.47)	16667 (12865 to 20282)	40.60 (31.33 to 49.21)	-30.88 (-41.62 to -20.36)	-4.29 (-4.5 to -4.09)
Tajikistan	5452 (4139 to 7090)	195.44 (147.43 to 255.05)	4824 (3374 to 6636)	83.00 (57.54 to 115.74)	-11.52 (-40.12 to 28.01)	-2.84 (-3.14 to -2.53)
Thailand	43150 (33526 to 55283)	124.44 (96.58 to 158.52)	64189 (48770 to 84832)	59.86 (45.53 to 79.40)	48.76 (9.69 to 104.11)	-2.82 (-2.98 to -2.66)
Timor-Leste	331 (232 to 440)	131.09 (92.51 to 176.08)	1120 (677 to 1673)	136.23 (82.89 to 204.05)	237.91 (117.80 to 405.37)	0.27 (0.11 to 0.44)
Togo	1825 (1383 to 2413)	146.00 (108.14 to 193.11)	4256 (2940 to 5985)	103.16 (70.28 to 143.98)	133.16 (67.87 to 223.97)	-1.29 (-1.45 to -1.14)
Tokelau	1 (1 to 2)	98.17 (67.62 to 132.36)	1 (1 to 1)	65.78 (49.99 to 91.24)	-28.71 (-47.39 to -0.83)	-1.53 (-1.62 to -1.44)
Tonga	48 (36 to 61)	89.29 (66.94 to 115.80)	53 (40 to 70)	66.80 (49.85 to 88.88)	11.10 (-16.31 to 44.57)	-1.07 (-1.22 to -0.93)
Trinidad and Tobago	1058 (859 to 1300)	124.61 (100.94 to 153.72)	964 (696 to 1301)	49.61 (35.94 to 67.04)	-8.96 (-31.52 to 19.84)	-3.45 (-3.74 to -3.17)
Tunisia	8986 (6378 to 11961)	193.48 (136.49 to 260.31)	16815 (11669 to 24010)	129.33 (89.91 to 183.79)	87.13 (34.10 to 159.60)	-1.53 (-1.66 to -1.39)

Location	1990		2021		1990-2021	
	DALYs Cases (95%UI)	ASMR (95%UI)	DALYs Cases (95%UI)	ASMR (95%UI)	Cases change, % (95%UI)	EAPC (95%CI)
Turkey	3901 (2771 to 4900)	189.87 (136.60 to 239.33)	7295 (4716 to 9810)	167.36 (108.40 to 228.42)	87.01 (41.44 to 145.17)	-0.55 (-1.08 to -0.01)
Turkmenistan	9 (7 to 12)	127.48 (93.58 to 169.67)	11 (8 to 15)	103.32 (77.57 to 136.67)	23.35 (-8.11 to 67.94)	-0.77 (-0.84 to -0.7)
Tuvalu	79084 (62308 to 96016)	225.10 (174.39 to 275.87)	68250 (51760 to 86659)	73.04 (55.00 to 93.29)	-13.70 (-31.67 to 8.11)	-4.11 (-4.35 to -3.87)
Uganda	2440 (1754 to 3542)	41.02 (29.29 to 59.85)	3689 (2609 to 5267)	26.30 (18.60 to 37.50)	51.15 (2.69 to 109.35)	-2.46 (-2.9 to -2.01)
Ukraine	199809 (165865 to 235608)	276.54 (229.29 to 326.50)	118180 (83373 to 159402)	159.35 (113.33 to 215.21)	-40.85 (-57.52 to -22.29)	-2.46 (-2.94 to -1.96)
United Arab Emirates	903 (656 to 1195)	151.80 (108.39 to 205.26)	3719 (2780 to 4793)	72.15 (52.49 to 97.03)	312.05 (216.40 to 439.40)	-1.68 (-2.09 to -1.26)
United Kingdom	143659 (117605 to 174807)	152.61 (125.69 to 184.64)	34343 (26154 to 44899)	26.18 (20.56 to 33.30)	-76.09 (-79.14 to -73.17)	-6.1 (-6.31 to -5.9)
United Republic of Tanzania	6959 (4947 to 9943)	68.13 (47.06 to 97.16)	14937 (9955 to 21640)	60.42 (39.96 to 89.92)	114.66 (32.58 to 239.11)	-0.8 (-0.94 to -0.65)
United States of America	244885 (201262 to 299873)	76.97 (63.36 to 93.93)	213391 (166723 to 272983)	38.99 (30.86 to 49.19)	-12.86 (-21.03 to -4.93)	-2.68 (-2.95 to -2.42)
United States Virgin Islands	33 (24 to 44)	38.23 (27.35 to 52.73)	31 (22 to 43)	17.84 (12.55 to 24.20)	-3.43 (-34.94 to 36.75)	-2.44 (-2.59 to -2.29)
Uruguay	4931 (4082 to 5971)	128.28 (106.77 to 154.28)	2752 (2216 to 3391)	53.31 (43.41 to 64.75)	-44.18 (-51.44 to -36.32)	-3.18 (-3.38 to -2.97)
Uzbekistan	11617 (9319 to 13888)	95.90 (76.50 to 115.35)	28981 (23111 to 35804)	100.74 (79.73 to 124.73)	149.47 (101.04 to 216.79)	-0.36 (-0.85 to 0.13)

Location	1990		2021		1990-2021	
	**DALYs Cases (95%UI)**	**ASMR (95%UI)**	**DALYs Cases (95%UI)**	**ASMR (95%UI)**	**Cases change, % (95%UI)**	**EAPC (95%CI)**
Vanuatu	71 (53 to 95)	106.86 (78.23 to 144.14)	129 (95 to 172)	69.55 (50.95 to 93.87)	82.43 (38.30 to 139.13)	-1.75 (-1.9 to -1.6)
Venezuela (Bolivarian Republic of)	4985 (3925 to 6020)	51.64 (40.03 to 63.38)	7197 (5113 to 9824)	24.40 (17.36 to 33.54)	44.38 (6.47 to 103.17)	-2.87 (-3.12 to -2.62)
Viet Nam	90097 (67437 to 119715)	229.26 (171.54 to 307.94)	220157 (156224 to 289890)	222.58 (158.79 to 293.05)	144.35 (67.37 to 247.49)	-0.04 (-0.14 to 0.06)
Yemen	13305 (9296 to 18709)	259.74 (180.98 to 367.45)	33772 (22121 to 46723)	228.07 (152.45 to 315.16)	153.83 (77.19 to 275.57)	-0.61 (-0.69 to -0.54)
Zambia	1753 (1210 to 2555)	69.95 (48.63 to 103.57)	3558 (2400 to 5099)	58.40 (38.28 to 83.18)	102.97 (37.60 to 194.34)	-0.97 (-1.12 to -0.82)
Zimbabwe	3263 (2481 to 4292)	89.47 (66.41 to 120.75)	6947 (5060 to 9279)	108.72 (78.43 to 144.84)	112.87 (50.06 to 196.73)	0.95 (0.45 to 1.44)

© 2024 Pang M. et al.
